# Identification and quantitation of multiple variants in RNA virus genomes

**DOI:** 10.1093/biomethods/bpae004

**Published:** 2024-02-03

**Authors:** Johnny Sena, Lovkesh Karwal, Callum Bell, Nicholas Devitt, Faye Schilkey, Claire Huang, Jill Livengood, Subash Das, Hansi J Dean

**Affiliations:** National Center for Genome Resources, Santa Fe, NM, United States; Vaccine Business Unit, Takeda Pharmaceuticals, Cambridge, MA, United States; National Center for Genome Resources, Santa Fe, NM, United States; National Center for Genome Resources, Santa Fe, NM, United States; National Center for Genome Resources, Santa Fe, NM, United States; Arboviral Diseases Branch, Division of Vector-Borne Diseases, Centers for Disease Control and Prevention, Fort Collins, CO, United States; Vaccine Business Unit, Takeda Pharmaceuticals, Cambridge, MA, United States; Vaccine Business Unit, Takeda Pharmaceuticals, Cambridge, MA, United States; Vaccine Business Unit, Takeda Pharmaceuticals, Cambridge, MA, United States

**Keywords:** RNA virus variant, dengue virus, PacBio, UMIs

## Abstract

The goal of the study was to identify and characterize RNA virus variants containing mutations spread over genomic distances >5 kb. As proof of concept, high-quality viral RNA of the Dengue 2 component of Takeda’s tetravalent dengue vaccine candidate (TDV-2) was used to develop a reverse transcription–polymerase chain reaction protocol to amplify a ∼5.3 kb cDNA segment that contains the three genetic determinants of TDV-2 attenuation. Unique molecular identifiers were incorporated into each viral cDNA molecule for PacBio library preparation to improve the quantitative precision of the observed variants at the attenuation loci. Following assay optimization, PacBio long-read sequencing was validated with multiple clone-derived TDV-2 revertant variants and four complex revertant mixtures containing various compositions of TDV-2 and revertant viruses. PacBio sequencing analysis correctly identified and quantified variant composition in all tested samples, demonstrating that TDV-2 revertants could be identified and characterized and supporting the use of this method in the differentiation and quantification of complex variants of other RNA viruses. Long-read sequencing can identify complex RNA virus variants containing multiple mutations on a single-genome molecule, which is useful for in-depth genetic stability and revertant detection of live-attenuated viral vaccines, as well as research in virus evolution to reveal mechanisms of immune evasion and host cell adaption.

## Introduction

RNA viruses have been recognized as important zoonotic agents originating from wildlife, occupying up to 44% of all emerging infectious diseases [1]. Viruses, which contain single-stranded positive-sense RNA (+RNA) genomes include both nonenveloped and enveloped viruses. Nonenveloped +RNA viruses that cause disease in humans comprise the families Picornaviridae, including polio, rhino, and hepatitis A viruses, Caliciviridae, including norovirus, and Hepeviride, including hepatitis E virus and Astroviridae [2]. Enveloped +RNA viruses pathogenic for humans comprise the family Flaviviridae, including yellow fever, dengue, West Nile, Japanese encephalitis, tick-borne encephalitis, Zika, and hepatitis C viruses, Togaviridae, including rubella and chikungunya viruses, and Coronaviridae, including severe acute respiratory syndrome viruses 1 and 2, middle east respiratory syndrome virus, and common cold coronaviruses.

The dengue virus (DENV) is an enveloped flavivirus with a diameter of 40–60 nm that contains a nucleocapsid associated with a linear positive-sense, 5′-capped RNA genome of approximately 11 kb in length that encodes three structural proteins (capsid, pre-membrane, and envelope) and seven nonstructural proteins (NS1, NS2a, NS2b, NS3, NS4a, NS4b, and NS5). The NS5 protein is the viral RNA-dependent RNA polymerase (RdRp) that amplifies RNA during viral replication [3]. Due to its lack of proofreading activity, RdRp is highly error-prone, exhibiting high mutation rates resulting in single-nucleotide polymorphisms (SNPs) in the genome, which drives rapid viral evolution, adaptation, and ultimately emergence of variants [3]. The positive selection of random mutations that arise due to error-prone RdRps allows RNA viruses to quickly evolve to escape host immune responses and/or antiviral treatments.

DENV circulates as four different serotypes, DENV-1 to -4, in tropical and sub-tropical areas worldwide and is transmitted to humans by mosquitoes. With an estimated 100–400 million annual infections, about half of the world’s population is at risk for DENV infection [4]. TAK-003 (Takeda Vaccines Inc.) is a live-attenuated tetravalent dengue vaccine (TDV) candidate that consists of an attenuated DENV-2 strain (TDV-2, clone-derived recombinant virus-based DENV-2 PDK-53 vaccine strain) and three attenuated and chimeric DENVs (TDV-1, -3, and -4) containing the prM and E protein genes of DENV-1, -3, and -4, respectively, expressed in the TDV-2 genome backbone [5–7]. There are nine nucleotide (nt) mutations between the genome of the parental wt DENV-16681 and PDK-53 (TDV-2) vaccine strains, and three loci were determined to be the main attenuation determinants [7, 8]. These three loci include a 5′-noncoding C to T transition at nt 57 (5′-NC-c57t), a G to A transition at nt 2579 (NS1-G53D), and an A to T transversion at nt 5270 (NS3-E250V) of the viral genome. Genetic attenuation of live viral vaccines renders the virus safe, while enabling sufficient replication to elicit protective immunity [9]. Live-attenuated virus vaccines, including TAK-003, generally contain multiple attenuating mutations to reduce the probability of a complete reversion to virulence. Frequently, these reversion loci are spread over long distances on the viral genome. Characterization of the genetic stability of attenuating mutations of live-attenuated virus vaccines is an important component of safety evaluation during vaccine production. Genetic stability studies have shown that the NS1-53D and NS3-250V loci in TDV-2 were highly stable, while 5′-NC-57t was more prone to reversion [9, 10]. Reversion of at least two of the three attenuation loci is normally required to reconstitute the wild-type phenotype, and the combination of the NS1-53 and NS3-250 attenuation loci was sufficient to retain all attenuation phenotypes characterized in mice [8] and mosquitoes [11].

Together, the three main attenuating mutations of TAK-003 span over 5 kb, or roughly half the total length of the viral genome. Few next-generation sequencing (NGS) methods are available to capture highly accurate genetic variants over a 5 kb distance based on a single molecule, long-read NGS. Illumina short-read sequencing is highly accurate (approximately one error per 1000 bp) [12] but is unable to phase multiple mutations spread over long distances. PacBio sequencing is characterized by long reads that can span distantly separated variants, but at the cost of having more frequent errors (the single-pass error rate of the PacBio RSII is 14%–15%). In practice, 3-fold Circular Consensus Sequencing (repetitive sequencing of the same template) reduces this to as low as 0.33% (Supplementary Table S1). Accordingly, PacBio RSII sequencing was a natural choice for characterizing multiple variants in a viral genome. Characterizing potential TAK-003 genetic variants in the three attenuation loci on single-viral genomes requires long-read sequencing of the cDNA library prepared from the nonpolyadenylated viral RNA. Pacific Biosciences of California, Inc. (PacBio) developed the first-generation RSII sequencer that uses single-molecule real-time (SMRT) technology for highly accurate long-read sequencing [13]. More recently, PacBio launched the second-generation Sequel System, which principally provides higher throughput sequencing at a lower running cost compared to the RSII [13].

Here, we describe the characterization of genetic variants of the three attenuation loci of TDV with high sequencing accuracy using a PacBio RSII sequencing method for nonpolyadenylated RNA templates, using Unique Molecular Identifiers (UMIs). UMIs reduce known sequencing errors such as preferential polymerase chain reaction (PCR) amplification of certain cDNA during library preparation. This method has application to the characterization of low-frequency variants of other +RNA viruses and attenuated viral vaccines.

## Materials and methods

### Cells and viruses

Vero cells were seeded in T25 cm^2^ flasks at 1.5 × 10^6^ cells/flask and grown overnight to 80%–90% confluency at 36 ± 2°C and 5% CO_2_ tension. The cells were observed for health and confluency under an inverted microscope prior to infection.

The wt DENV-2 16681 and a clone-derived laboratory stock of TDV-2 vaccine equivalent, D2/IC-VV45R (referred to as TDV-2 in this article), and the vaccine revertant variants, including D2/IC-P1 (A to G at nt 2579, NS1-53G reversion), P3 (T to A at nt 5270, NS3-250E reversion), P5 (T to C at nt 57, 5′-NC-57c reversion), P51 (contains 5′NC-57 and NS1-51 reversions), and P513 (contains all three reversions at 5′-NCR-57, NS1-250, and NS3-250) were previously generated and provided by Dr Huang at the CDC [7]. The low-cell passaged CDC stocks were diluted in Dulbecco’s Modified Eagle Medium containing 1% fetal bovine serum and 1% penicillin–streptomycin and administered to Vero cells at 0.1 multiplicity of infection to prepare stocks for the study. The virus was allowed to adsorb to the cells in culture for 1 h at 37°C and a CO_2_ concentration of 5%. After incubation, the inoculum was aspirated, and cells were replenished with 6 ml of fresh culture medium and incubated for an additional 5–7 days. The cell culture medium was replaced on day 5. On day 7, the cell culture medium was centrifuged at 10 000*g* for 10 min to remove cellular debris at 4°C and the virus-containing supernatant was collected.

### Preparation of complex revertant mixtures

Four complex revertant mixture pools (Complex Mix 1–4) were prepared by mixing TDV-2, various vaccine revertants, and/or wt DENV-2 16681. The expected (targeting) percentage of virus genome composition in each mixture was based on the genome copies of individual virus determined by reverse transcription-quantitative PCR prior to mixing, and the mixtures were sent to the National Center for Genome Resources (NCGR) for processing without disclosing any information on the composition (blind to NCGR). Detailed composition of the complex mixtures and the expected reversion sites are shown in Supplementary Table S2.

### Viral RNA extraction

RNA was extracted from 140 μl of virus-infected Vero cell culture supernatant using the Omega Viral RNA extraction kit according to the manufacturer’s instructions. The RNA yield was measured on a Nanodrop 2000 spectrophotometer (Thermo Fisher Scientific). RNA with 260/280 ratios of approximately 1.8–2.0 and containing at least 1 × 10^8^ viral genome copies, as determined by reverse transcription (RT)–qPCR, was sent to NCGR and stored below −65°C until used for RT–PCR.

### Quantification of viral RNAs using RT–qPCR

The RT–qPCR was performed in a 25 μl volume containing 6.25 μl of 4X TaqMan Fast Virus 1-Step Master Mix (Thermo Fisher Scientific), 0.5 μl of 20 μM forward and reverse primers, 0.5 μl of 20 μM probes, 5 μl of viral RNA being tested, and 12.5 μl RNAse-free water using the QuantStudioTM 7 Flex System (Applied Biosciences). The primers (D2-1929, cD2-2116) and probe (D2-2000VIC-MBG/NFQ quencher) anneal at envelope (E) gene of the DENV-2 and their sequences have been reported previously [10, 14]. The viral copy number was determined using a standard curve generated by an in-vitro transcribed DENV-2 RNA as previously described [10]. The concentration of standard RNA was determined based on the Certificate of Analysis received from Pharmaceutical Product Development, a global contract research organization. Briefly, a high concentration stock of DENV-2 RNA transcript obtained from CDC was tested by droplet digital PCR and was further diluted and quantified by RT–qPCR against standards previously measured by Ribogreen assay (Thermo Fisher Scientific) to make stocks at 2.7 × 10^7^ copies/μl. The stock was 10-fold serially diluted to generate the standard curve in the RT–qPCR to measure the concentration of the samples.

### RT–PCR and nested PCR

The regions surrounding the attenuation sites at positions 57 and 5270 in the TDV-2 genome (GenBank U87412.1) were analyzed using mfold software to identify regions with minimal secondary structure [15]. Segments with un-duplexed regions were chosen and six primer pairs were selected (Supplementary Table S3) to amplify these segments using the Primer-Blast server at NCBI with the “Specificity Check” parameter disabled [16]. Primers were purchased from Integrated DNA Technologies, lyophilized with standard desalting buffer, and resuspended in PCR grade water at 100 μM. The primer stocks were stored at below −20°C and then diluted to 10 μM prior to use in RT–PCR. Details of the RT–PCR and nested PCR are described in the supplementary methods.

### RT–PCR optimization to amplify long cDNA fragments with high specificity

To identify new combinations of PCR primers for optimization, electronic PCR (ePCR) with the previously designed primer sets (Supplementary Table S3) was performed, allowing for 30% primer binding site mismatch, with Unipro UGENE software [17]. Based on the ePCR analysis that demonstrated low “off target” binding and results of nested PCR, primers 5F and 3R were selected for optimization.

Reverse transcription was performed using viral RNA (∼10^8^ viral copies) and the “First-Strand synthesis” kit from Thermo Fisher Scientific with primer 3R at two different reaction temperatures (50°C and 55°C). Touchdown PCR was conducted with primers 5F and 3R as described in the supplementary methods. The PCR product was analyzed with a 1% TAE agarose gel stained with ethidium bromide using electrophoresis.

### Introduction of UMIs into RT–PCR amplicons

A fully random 16-nt UMI and a 5′-PCR Primer IIA binding site were added to the 5′-end of the 3R primer (Supplementary Table S3). RT was performed with the UMI 3R primer with about 10^8^ copies of viral RNA using the Superscript III reaction kit (Thermo Fisher Scientific). PCR was performed with the 5F and 5′-PCR Primer IIA primers using Advantage 2 polymerase mix (Clontech Lab 3P). The details of the RT–PCR protocol are provided in the supplementary methods.

### PacBio library preparation

The PCR reaction with UMI was scaled up 3-fold and cleaned with Ampure XP beads as described in the supplementary methods. An aliquot was analyzed with 1% TAE agarose gel and used for fragment analysis by the Bioanalyzer 2100 with the Agilent DNA 12000 Kit. The remaining product was used to construct a PacBio sequencing library using PacBio’s amplicon library preparation protocol and a 1 μl aliquot of the library was used for DNA fragment size analysis by the Bioanalyzer 2100 with the Agilent DNA 12000 Kit.

### PacBio sequencing and variant analysis

PacBio sequencing libraries of TDV-2, revertants (P1, P3, P5, and P51), and revertant complex mixture pools (Complex Mix 1 − 4) were constructed by PacBio amplicon library preparation as described above with additional details provided in the supplementary methods. The protocol included sequencing to confirm the TDV-2 and revertant sequences. One SMRTcell was sequenced per library sample, and variant analysis was done as described in the supplementary methods.

### Quantification of variants by read-based and UMI-based methods

This workflow consisted of trimming off the SMARTER UMI 3R adapter sequence, extracting the UMI sequence and appending it to the read ID of the TDV-2 insert fastq file, aligning the trimmed fastq file to the DENV-2 PDK-53 reference genome (GenBank U87412.1) using BWA-mem [18] and counting the variants at each attenuation site.

## Results

### Assay development and optimization

In the initial stages of assay development, six RT–PCR primer pairs were designed to evaluate regions surrounding the attenuation sites of the TDV-2 (Supplementary Table S3). Reverse transcription was performed on the laboratory stock of TDV-2 (D2/IC-VV45R [9]) RNA using the reverse primer of each primer pair, followed by PCR. The results indicated that amplicons of 5.3 kb that encompass all three attenuation loci could be obtained (Supplementary Figure S1A). However, many nonspecific amplicons were also observed. To reduce nonspecific PCR amplification, RT–PCR products generated from primer sets 1 and 4 were diluted and used for nested PCR with primers 5F and 3R for various reaction cycles. A total of 11 nested PCR cycles was considered optimal for subsequent large-scale nested PCR since it produced the lowest amount of nonspecific products (Supplementary Figure S1B). The optimal nested PCR, using RT–PCR product 1, was scaled up 3-fold to be used for PacBio sequencing library construction. Fragment analysis of the library showed a product of the expected size (5995 bp) and a nonspecific product of 3368 bp (Supplementary Figure S2). The library was sequenced in one SMRT cell on the PacBio RSII, the circular consensus sequence (CCS) reads aligned to the TDV-2 reference genome and variants were quantified. The results showed that most reads (96.7%) contained the expected nucleotides, T-A-T at the three attenuation loci 57, 2579, and 5270, respectively. Other combinations of variants (total 2.64%) were observed including the wild-type reversion at one or two attenuation sites (Supplementary Table S4).

**Figure 1.**
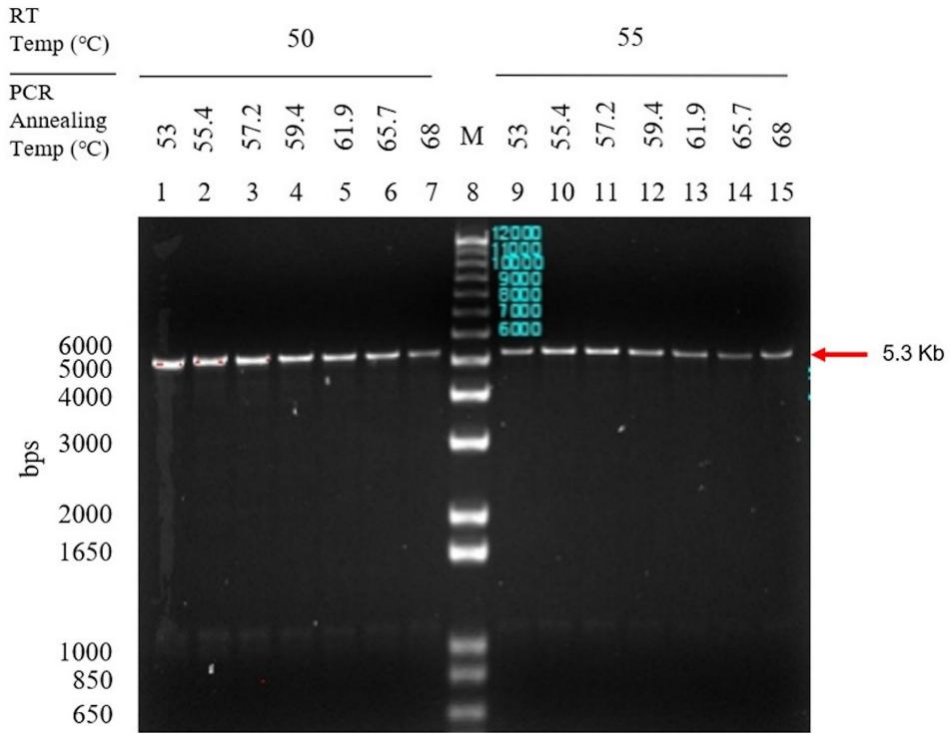
RT and PCR temperature optimization to amplify TDV-2 cDNA using primers 5F and 3R.

Our initial development result demonstrated that the PacBio deep sequencing would be an appropriate platform to identify multiple SNPs across >5 kb distance in a single-genome molecule. However, since the process included the nested-PCR that could potentially introduce additional PCR amplification bias and errors prior to PacBio library preparation, we further optimized the long-fragment RT–PCR for higher specificity to eliminate the additional nested-PCR requirement. A new combination of primers (5F and 3R) was identified using ePCR that demonstrated low “off target” binding. The RT of TDV-2 RNA with primer 3R was tested at 50 or 55°C followed by PCR with primers 5F and 3R at gradient elongation temperatures of 53–68°C. The results showed strong amplification of a clear band above 5 kb and a faint band around 1 kb (Figure 1). The optimal RT and PCR elongation temperatures were determined to be 55 and 57°C, respectively (well 11). The nonspecific faint band was particularly notable at the lower RT temperature (wells 1–7).

**Figure 2.**
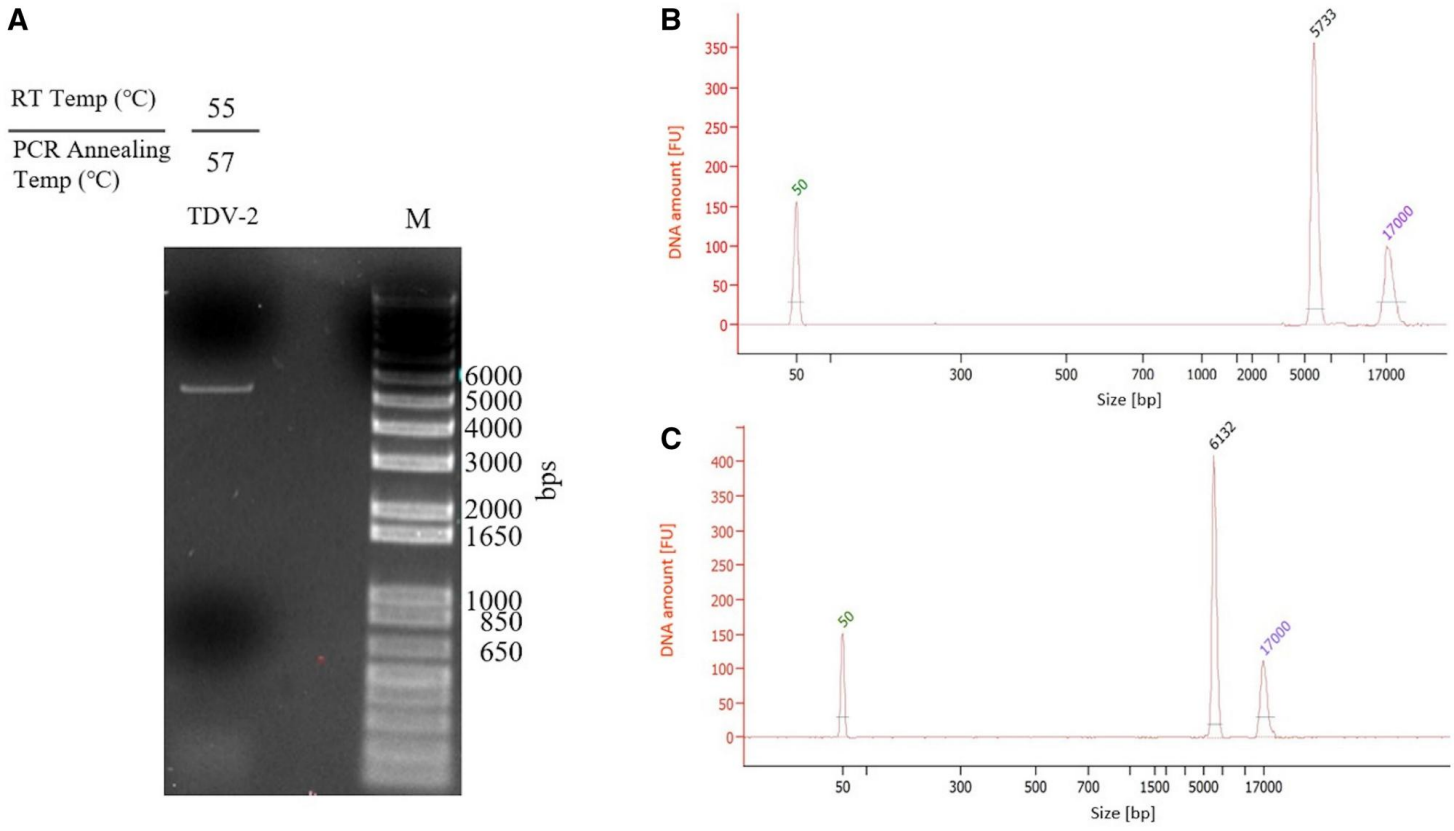
Optimized RT–PCR protocol applied to TDV-2. (A) RT–PCR of TDV-2 RNA using optimized RT and PCR annealing temperatures. (B) Bioanalyzer electropherogram plot of scaled up PCR reactions. (C) Bioanalyzer electropherogram plot of TDV-2 PacBio library. The 50 and 17 000 bp peaks in the electropherogram plots are the kit markers. Abbreviation: FU, fluorescent unit.

To further incorporate UMIs into the cDNA library for PacBio sequencing analysis using UMI read, we further added random 16-nt UMIs and a 5′-PCR Primer IIA primer binding site to the 5′-end of the 3R primer (UMI 3R in Supplementary Table S3) for the RT–PCR. RT–PCR was performed with 5F and UMI 3R primers to amplify the viral cDNA. The RT–PCR resulted in a single amplification product of the expected size, between 5 and 6 kb (Figure 2A and B). Fragment analysis of an aliquot of the PacBio cDNA library made from the UMI-incorporated RT–PCR product demonstrated a clean library without off-target products (Figure 2C). The library was sequenced in one SMRT cell on the PacBio RSII, the CCS reads were aligned to the TDV-2 reference genome, and variants at the attenuation sites of interest were quantified using read and UMI counts. Generally, variant counts using UMIs decreased compared to read counts (Table 1); however, the overall proportion of variants detected was similar between the two quantification methods.

**Table 1.** Quantification of TDV-2 variants using read counts and UMIs.

Attenuation Sites 57—2579—5270	Read count	Read percentage	UMI count	UMI percentage
**T—A—T^a^**	7041	96.175	6698	96.153
**T—G—T**	184	2.513	177	2.541
**T—A—G**	33	0.451	31	0.445
**G—A—T**	18	0.246	17	0.244
**C—A—T**	14	0.191	13	0.187
**A—A—T**	9	0.123	8	0.115
**T—A—C**	8	0.109	8	0.115
**T—C—T**	6	0.082	6	0.086
**T—A—A**	3	0.041	3	0.043

a Black: TDV-2 nucleotide, blue: wild-type DENV-2 16681 nucleotide, red: unknown significance. Abbreviations: TDV-2, VV45R laboratory derived tetravalent dengue vaccine equivalent; UMI, unique molecular identifier.

### PacBio sequencing of revertant viruses and complex mixture pools

Sanger sequencing was performed to confirm the clone-derived TDV-2 revertant virus stocks, P1, P3, P5, and P51, which were amplified once in Vero cells from the CDC low-cell passaged virus seeds. The primers (Supplementary Table S5) used for the RT–PCR and sequence were based on the previously established protocol at the CDC laboratory [9]. The results showed RT–PCR amplicons of the expected size (Supplementary Figure S3A) with expected reversions present in the four revertant stocks (Supplementary Figure S3B). To mimic manufacturing in-process samples that could contain subpopulations of revertant variants, four complex mixture pools (Complex Mix 1–4) were prepared by combining the confirmed revertant viruses and the TDV-2 in various proportions of each virus (Supplementary Table S2). These complex mixes were blinded prior to being submitted for the PacBio sequencing methods described above.

**Figure 3.**
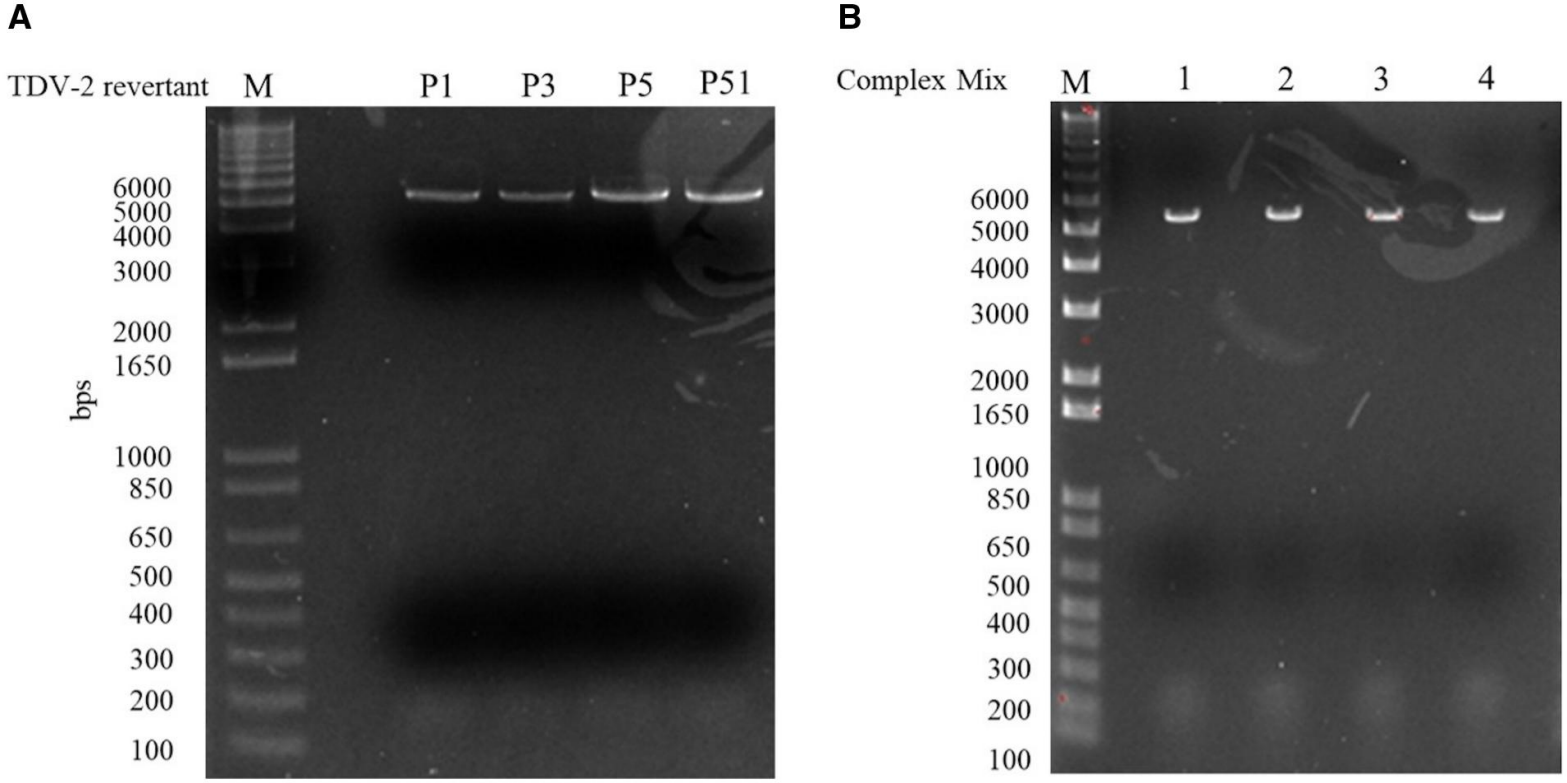
Optimized RT–PCR of (A) TDV-2 revertant and (B) four complex revertant mixtures (lanes 1–4).

The optimized long-fragment RT–PCR successfully produced a single cDNA fragment of expected size for each revertant virus and Complex Mix (Figure 3). Fragment analysis, following library preparation, confirmed that a uniform 6 kb cDNA fragment library was generated for each revertant and Complex Mix (Supplementary Figures S4 and S5). One or two SMRT cells per library were sequenced on the PacBio RSII instrument, the CCS reads aligned to the reference genome, and variants quantified using read and UMI counts. The deep sequencing outcome of TDV-2 revertants validated reversions at the expected position(s) that were the most abundant (98.6%–99.6%) (Supplementary Table S6).

The PacBio sequence results of the four Complex Mix samples also demonstrated the successful identification of all viruses mixed in each sample. The percentage of the most abundant variants detected largely agreed with the expected compositions based on the qRT–PCR genomic equivalent concentration of each virus during mixture preparation (Table 2 and Supplementary Table S2). In Complex Mix 1, 13 variants at positions 57, 2579, and 5270 were identified with T-A-A (as P3) being the most abundant, followed by T-G-T (as P1), T-A-T (as TDV-2), and C-A-T (as P5). The remaining variants made up a very small percentage of the population (0.87%), with variants as low as 0.012% detected by UMI count. Complex Mix 2 contained the largest number (18) of variants detected, with C-G-A (P513) being the most abundant followed by T-A-T (TDV-2), C-G-T (P51), and T-A-A (P3). The remaining 14 variants made up 0.84% of the population, with the lowest variants detected at 0.015%. Complex Mix 3 contained the fewest (11) variants among the four mixtures. Variant C-G-A (P513) was the most abundant, followed by C-G-T (P51) and T-A-A (P3), with the remaining eight variants making up 0.37% of the population and detecting variants as low as 0.012%. Fourteen variants were detected in complex Mix 4; however, two variants, C-G-A (P513) and T-A-T (TDV-2) made up 99.3% of the population, with the remaining variants making up only 0.7% of the population, including those being detected at a very low level (0.013%). When comparing the percentages of viral genomes added in the mixtures, the average absolute difference between the expected and the detected genomes using UMIs was approximately 4.4, with absolute differences ranging from 0.38 to 11.73 (Table 3).

**Table 2.** Complex mixtures 1–4 read and UMI counts.

Revertant	Attenuation sites 57—2579—5270	Read count	Read percentage	UMI count	UMI percentage
Complex Mix 1	**T—A—A** ^a^ **(P3)**	5261	57.781	4955	57.764
	**T—G—T (P1)**	2117	23.251	1996	23.269
	**T—A—T (P513)**	1025	11.258	961	11.203
	**C—A—T (P5)**	625	6.864	591	6.890
	**T—G—A**	43	0.472	42	0.490
	**C—G—T**	9	0.099	9	0.105
	**C—A—A**	8	0.088	8	0.093
	**T—A—G**	8	0.088	7	0.082
	**C—A—C**	3	0.033	3	0.035
	**G—A—A**	3	0.033	3	0.035
	**A—G—T**	1	0.011	1	0.012
	**T—T—A**	1	0.011	1	0.012
	**T—T—T**	1	0.011	1	0.012
Complex Mix 2	**C—G—A (P513)**	2663	37.141	2558	37.164
	**T—A—T (TDV-2)**	2119	29.554	2037	29.595
	**C—G—T (P51)**	1547	21.576	1484	21.560
	**T—A—A (P3)**	780	10.879	746	10.838
	**T—G—T**	17	0.237	16	0.232
	**T—G—A**	11	0.153	11	0.160
	**C—A—T**	9	0.126	9	0.131
	**C—A—A**	8	0.112	6	0.087
	**T—A—G**	5	0.070	5	0.073
	**C—G—C**	2	0.028	2	0.029
	**C—T—T**	2	0.028	2	0.029
	**A—A—T**	1	0.014	1	0.015
	**C—G—G**	1	0.014	1	0.015
	**G—A—A**	1	0.014	1	0.015
	**G—A—T**	1	0.014	1	0.015
	**T—A—C**	1	0.014	1	0.015
	**T—C—T**	1	0.014	1	0.015
	**T—T—T**	1	0.014	1	0.015
Complex Mix 3	**C—G—A (P513/wt)**	5740	64.756	5428	64.889
	**C—G—T (P51)**	2237	25.237	2101	25.117
	**T—A—A (P3)**	853	9.623	805	9.623
	**C—A—A**	13	0.147	12	0.143
	**C—G—C**	5	0.056	5	0.060
	**C—G—G**	5	0.056	4	0.048
	**T—G—A**	4	0.045	4	0.048
	**A—G—A**	2	0.023	2	0.024
	**T—A—T**	2	0.023	2	0.024
	**T—G—T**	2	0.023	1	0.012
	**C—A—T**	1	0.011	1	0.012
Complex Mix 4	**C—G—A (wt)**	5159	65.644	4926	65.575
	**T—A—T (TDV-2)**	2645	33.656	2532	33.706
	**C—A—T**	11	0.140	11	0.146
	**C—G—T**	9	0.115	9	0.120
	**T—G—A**	8	0.102	8	0.106
	**C—A—A**	6	0.076	6	0.080
	**T—A—A**	5	0.064	5	0.067
	**C—G—G**	4	0.051	4	0.053
	**T—A—C**	4	0.051	3	0.040
	**T—G—T**	3	0.038	3	0.040
	**T—A—G**	2	0.025	2	0.027
	**A—G—A**	1	0.013	1	0.013
	**C—T—A**	1	0.013	1	0.013
	**T—C—T**	1	0.013	1	0.013

a Black: vaccine nucleotide, blue: wild-type nucleotide, red: unknown significance.

**Table 3.** Proportion of TDV-2, revertants, and DENV-2 in complex mixtures using UMIs.

RNA	Complex Mix 1			Complex Mix 2			Complex Mix 3			Complex Mix 4		
	UMI^a^ (%)	Expected (%)	Difference (|E-U|)^b^	UMI (%)	Expected (%)	Difference (|E-U|)^b^	UMI (%)	Expected (%)	Difference (|E-U|)^b^	UMI (%)	Expected (%)	Difference (|E-U|)^b^
P1	23.27	35	11.73	NA	NA	NA	NA	NA	NA	NA	NA	NA
P3	57.76	50	7.76	10.84	10	0.84	9.62	10	0.38	NA	NA	NA
P5	6.89	5	1.89	NA	NA	NA	NA	NA	NA	NA	NA	NA
P51	NA	NA	NA	21.56	20	1.56	25.12	20	5.12	NA	NA	NA
P513	NA	NA	NA	37.16	40	2.84	64.89^c^	40	5.11	NA	NA	NA
wt DENV-2	NA	NA	NA	NA	NA	NA		30		65.57	75	9.43
TDV-2	11.2	10	1.2	29.59	30	0.41	NA	NA	NA	33.7	25	8.7

a Determined by UMI.

b Absolute value of Expected % − UMI%.

c P513 and WT DENV-2 have the same nucleotides at positions 57, 2579, and 5270 and were indistinguishable.

NA, not applicable.

Utilizing CCS read correction resulted in the error rates for TDV-2 being <2%; while the error rates for revertant and complex mixture samples were <1% (Supplementary Table S1). Interestingly, when assessing the relative percentage of variant combinations within a sample, counting variant combinations using UMIs did not significantly impact the relative proportions of variant combinations identified versus counting reads alone. Overall, the workflow and PacBio sequencing method described here could accurately identify and quantify combinations of variants at attenuation loci in TAK-003 RNA genomes.

## Discussion

In this study, we demonstrated that PacBio sequencing can detect genomic variants in complex mixtures of clone-derived viruses containing substitutions at one, two, or three loci over a ∼5 kb span on the same RNA molecule. The long-read lengths described here can capture and quantitatively distinguish variants at attenuation loci that are spread over long distances with good accuracy. This approach can therefore overcome the primary limitation of short-range sequencing (being unable to detect multiple mutations spread over 1 kb in a single-genome molecule), simultaneously reducing the high error typically associated with the PacBio RSII by employing circular consensus sequencing.

Quantification of genome variants using PCR amplification as part of the process suffers from uncertainty introduced by PCR amplification bias. UMIs reduce this problem by establishing a unique identity for each input molecule and have become standard in RNA-Seq workflows where they improve the accuracy of digital gene expression analysis. UMIs have also found utility in virology to distinguish rare sequence variants from PCR and sequencing errors [19]. More recently, high-throughput amplicon sequencing approaches combining UMIs with Oxford Nanopore Technologies or PacBio circular consensus long-read sequencing were used to generate high-accuracy single-molecule consensus sequences with mean UMI consensus error rates of <0.01% [20]. Furthermore, PacBio’s Revio long-read sequencer is also known to have higher throughput and shorter run times compared to the Sequel IIe system, with a comparable error rate of <0.1% [21].

It is important to note that quantification of cDNA was not done prior to PCR in this instance. Alternative methods that incorporate UMIs into cDNA by PCR [22] can result in UMI collisions if too many cDNAs are added to the PCR reaction. However, UMI collisions cannot occur during PCR of the cDNA because the UMIs are introduced as part of the primer during first-strand cDNA synthesis. Thus, UMI collisions can only occur during first-strand cDNA synthesis. The number of viral copies was ∼10^8^ viral RNA copies/μl of RNA as determined by RT-qPCR. The amount of RNA used per RT reaction was 5 − 6.5 µl. Thus, 6.5 µl × 10^8^ viral RNA copies/µl = ∼6.5 × 10^8^ viral copies/RT reaction. Random 16-mer UMIs number 4^16^ unique UMI sequences, equating to a 6.6-fold excess. Given that the efficiency of reverse transcription is certain to be <100%, the actual excess is even greater. UMI collisions are inevitable, reducing their probability is possible by increasing the length of the UMI, but this also increases the likelihood of sequencing errors in the UMI. In our analysis, sequencing errors in the UMI were mitigated by merging UMIs separated by one mismatch.

The PacBio RSII records the incorporation of nucleotides into a single-DNA template molecule by an immobilized DNA polymerase and involves circularization of the template allowing multiple sequencing passes of the same molecule [23]. A CCS can be generated from multi-pass reads that eliminate most of the sequencing errors since they are randomly distributed along the read sequence [23, 24]. In fact, the error rates for TDV-2 were <2%, and the error rates for revertant and complex mixture samples were <1% using CCS read correction (Supplementary Table S1).

When comparing the number of variant combinations in TDV-2 using the initial quantification pipeline done with BLASR and Quiver that identified 15 variant combinations at the attenuation sites of interest (Supplementary Table S4), the UMI quantification method identified 9 variant combinations (Table 1). This discrepancy potentially could be explained by the fact that nested PCR was performed in the original protocol, which could result in additional PCR errors, these experiments cannot be directly compared. Alternatively, this might be due to 35% more sequencing yield in the initial protocol resulting in more variants being detected, or to the slightly higher error rate in the initial protocol that could lead to increased false positives (Supplementary Figure S5).

To mimic samples and circumstances where vaccine virus attenuation loci have reverted to wild-type, the optimized RT–PCR, library preparation, and variant detection protocols were applied to four complex mixtures containing various combinations of revertant viruses, wt DENV-2, and/or TDV-2 to quantify the variants based on nt 57, 2579, and 5270 in each mixture. As expected, the UMI variant counts were less than counts without using UMIs in all four complex mixes, presumably by reducing duplicate count error. However, when assessing the relative percentage of variant combinations within a sample, counting variants using UMIs did not significantly change the outcomes identified by counting reads alone. This suggests that standard PacBio sequencing, without UMIs, may be sufficient to determine the relative percentage of variant combinations within a sample, at least with the number of PCR cycles and sequencing depth used in these experiments. On the other hand, if absolute quantification of variants is required then the use of UMIs for improved accuracy may be useful.

The methods developed in this study could be adapted to amplify the entire dengue viral genome when full genome sequencing is required, such as studies of quasi-species dynamics. Furthermore, these protocols can extend to clinical diagnostics and good laboratory practice conditions where clinical serum samples can be directly tested for the presence of viral genomes. With proper positive control plasmid or cDNA, the limit of detection can be established, which could be useful in assessing virus load. The methods can also be applied to epidemiology studies and the detection of emergent viral variants. Human RNA viruses are prone to genomic mutations that help them adapt to host and environmental pressures, as well as facilitate zoonotic and/or vector infection and transmission cycles. Fast, accurate, and specific long-range sequencing methods can therefore help to detect emerging virus variants and provide data to better identify critical genetic alterations involved in immune evasion and host species adaption that allows vector or zoonotic transmission of novel viruses to human. During the preparation of this article, a similar PacBio CCS sequencing method incorporating UMIs accurately sequenced the 6.1 kb severe acute respiratory syndrome coronavirus 2 surface protein gene region from large numbers of individual virus genomes and revealed spike protein variants coincident with mounting humoral immunity during acute coronavirus disease 2019 [25].

In conclusion, we accurately identified and quantified multi-SNPs at attenuation loci over a 5 kb span in the viral genome of a live-attenuated dengue vaccine virus using a combination of UMIs with PacBio sequencing to develop an accurate deep sequence and bioinformatics workflow. Variants were detected within a complex mixture of genomes without the need for extensive bioinformatics approaches. Accurate detection of reversions at attenuation loci of live-attenuated vaccine viruses is important for quality control analyses to ensure that reversion is limited during the vaccine manufacturing process and for vaccine safety analyses.

## Supplementary Material

bpae004_Supplementary_Data

## Data Availability

The data underlying this article are available in the article and in its online supplementary material.
