# Supplementary material for: Identification and quantitation of multiple variants in RNA virus genomes
Source: Biol Methods Protoc. 2024 Feb 3;9(1):bpae004. doi: 10.1093/biomethods/bpae004 (PMC10898329; doi:10.1093/biomethods/bpae004)

**Supplementary tables**

Supplementary Table 1: PacBio sequencing statistics for TDV-2, revertants and complex mixtures

| **Sample** | **Yield (Mbps)** | **Reads of insert** | **Mean number of passes** | **Mean read of insert length (bps)** | **Mean read quality (%)** | **Mean read error (100 - mean read quality %)** |
| --- | --- | --- | --- | --- | --- | --- |
| TDV-2 Nested PCR | 66.3 | 12,467 | 6.15 | 5321 | 98.28 | 1.72 |
| TDV-2 with UMI | 42.9 | 8038 | 4.99 | 5346 | 98.38 | 1.62 |
| P1 | 81.9 | 17540 | 10.15 | 4669 | 99.35 | 0.65 |
| P3 | 52 | 11134 | 10.56 | 4674 | 99.48 | 0.52 |
| P5 | 66 | 14072 | 9.24 | 4699 | 99.24 | 0.76 |
| P51 | 65.6 | 12199 | 8.67 | 5378 | 99.55 | 0.45 |
| Complex Mix 1 | 52.3 | 9737 | 8.57 | 5375 | 99.51 | 0.49 |
| Complex Mix 2 | 44.5 | 8959 | 9.5 | 4967 | 99.61 | 0.39 |
| Complex Mix 3 | 49.7 | 9254 | 9.12 | 5380 | 99.6 | 0.4 |
| Complex Mix 4 | 44.1 | 8195 | 8.95 | 5388 | 99.67 | 0.33 |

Supplementary Table 2: Composition of complex revertant mixtures

| **RNA Sample** | **Complex mix 1** | **Complex mix 2** | **Complex mix 3** | **Complex mix 4** | **Nucleotide position of reversion** | **Reversion site** | **Number of reversion sites** |
| --- | --- | --- | --- | --- | --- | --- | --- |
|  | **Expected composition (%)** | **Expected composition (%)** | **Expected composition (%)** | **Expected composition (%)** |  |  |  |
| P1 | 35 | NA | NA | NA | 2579 | NS1 | 1 |
| P3 | 50 | 10 | 10 | NA | 5270 | NS3 | 1 |
| P5 | 5 | NA | NA | NA | 57 | 5 ' Non-coding Region | 1 |
| P51 | NA | 20 | 20 | NA | 57, 2579 | 5 ' Non-coding Region, NS1 | 2 |
| P513 | NA | 40 | 40 | NA | 57, 2579, 5270 | 5 ' Non-coding Region, NS1, NS3 | 3 |
| WT DENV-2 | NA | NA | 30 | 75 | NA* | NA* | NA* |
| TDV-2 | 10 | 30 | NA | 25 | NA | NA | 0 |

*Nucleotides at positions 57, 2579, 5270 are the same as P513 but are not considered reversions as they are the wild-type for DENV-2

Abbreviations: WT DENV, wild-type dengue virus; TDV-2, Tetravalent Dengue Vaccine-2 equivalent (VV45R)

Supplementary Table 3: Reverse transcription and PCR primers used for RT-PCR optimization

| **Name** | **Direction** | **Sequence** | **Start** | **Stop** | **Tm** | **Product** |
| --- | --- | --- | --- | --- | --- | --- |
| 1F | Forward | AAGACAGATTCTTTGAGGGAGCTA | 26 | 49 | 59 | 5368 |
| 1R | Reverse | GGTCTGTGAAATGGGCTTCG | 5393 | 5374 | 59 |  |
| 2F | Forward | GACAGATTCTTTGAGGGAGCTAA | 28 | 50 | 58 | 5367 |
| 2R | Reverse | GGGTCTGTGAAATGGGCTTC | 5394 | 5375 | 59 |  |
| 3F | Forward | ACAGATTCTTTGAGGGAGCTAAG | 29 | 51 | 58 | 5364 |
| 3R | Reverse | GTCTGTGAAATGGGCTTCGTC | 5392 | 5372 | 60 |  |
| 4F | Forward | AGACAGATTCTTTGAGGGAGCTAAG | 27 | 51 | 60 | 5366 |
| 4R | Reverse | GTCTGTGAAATGGGCTTCGT | 5392 | 5373 | 58 |  |
| 5F | Forward | GACAGATTCTTTGAGGGAGCTAAG | 28 | 51 | 59 | 5373 |
| 5R | Reverse | CTTGCTGGGTCTGTGAAATGG | 5400 | 5380 | 59 |  |
| 6F | Forward | AAGACAGATTCTTTGAGGGAGCTAA | 26 | 50 | 60 | 5370 |
| 6R | Reverse | TGGGTCTGTGAAATGGGCTTC | 5395 | 5375 | 61 |  |
| 5F | Forward | GACAGATTCTTTGAGGGAGCTAAG | 28 | 51 | 59 | 5405 |
| 5' PCR Primer IIA | Reverse | AAGCAGTGGTATCAACGCAGAGTAC | 28 | 51 | 59 |  |
| UMI 3R | 3’ RT Oligo containing UMI | AAGCAGTGGTATCAACGCAGAGTACNNNNNNNNNNNNNNNNGTCTGTGAAATGGGCTTCGTC |  |  |  |  |

Abbreviations: PCR, polymerase chain reaction; RT, reverse transcription; Tm, melting temperature; UMI, unique molecular identifiers

Supplementary Table 4: Quantification of TDV-2 variants using read counts

| **Attenuation sites 57 - 2579 - 5270** | **Read counts** | **Read percentage** |
| --- | --- | --- |
| T - A - T | 10472 | 96.703 |
| T - G - T | 201 | 1.856 |
| T - A - A | 48 | 0.443 |
| C - A - T | 28 | 0.259 |
| T - A - C | 21 | 0.194 |
| T - A - G | 17 | 0.157 |
| A - A - T | 15 | 0.139 |
| G - A - T | 12 | 0.111 |
| T - C - T | 5 | 0.046 |
| T - G - A | 4 | 0.037 |
| C - G - T | 2 | 0.018 |
| A - G - T | 1 | 0.009 |
| T - G - G | 1 | 0.009 |
| C - A - C | 1 | 0.009 |
| A - A - C | 1 | 0.009 |

*Black: vaccine nucleotide, blue: wild-type nucleotide, red: unknown significance

Supplementary Table 5: Primers for RT-PCR and sanger sequencing*

| **Primer** | **Direction** | **Sequence** | **Start** | **Stop** | **Nucleotide position of reversion** | **Reversion region** |
| --- | --- | --- | --- | --- | --- | --- |
| D2-1 | Forward | AGTTGTTAGTCTACGTGGACCGAC | 1 | 24 | 57 | 5’ Non-coding region |
| D2-2389 | Forward | GTGACACTGTATTTGGGAGTCATGGTGCAG | 2389 | 2418 | 2579 | NS1 |
| D2-4996 | Forward | GGTAATGGTGTTGTTACAAGGAGTG | 4996 | 5020 | 5270 | NS3 |
| D2-5219 | Forward | AAGCCCTTAGAGGACTTCCAATAA | 5219 | 5242 | 5270 | NS3 |
| cD2-373 | Reverse | TCAGCATCCTTCCAATCTCTTTCC | 373 | 350 | 57 | 5’ Non-coding region |
| cD2-2736 | Reverse | CCGCAGAGATCGTTTTCCTGCCTG | 2736 | 2713 | 2579 | NS1 |
| cD2-5318 | Reverse | CTAACTGGTGATAGCAGCCTCATGG | 5318 | 5294 | 5270 | NS3 |
| cD2-5358 | Reverse | GAAATGGGCTTCGTCCATGATAATCAGG | 5358 | 5331 | 5270 | NS3 |

The above primers were kindly provided by the CDC (Fort Collins, CO)

Supplementary Table 6: Quantification of TDV-2 revertants using read and UMI counts

| **Revertant** | **Attenuation site 57 - 2579 - 5270** | **Read count** | **Read percentage** | **UMI count** | **UMI percentage** |
| --- | --- | --- | --- | --- | --- |
| P1 | **T - G - T** | 11993 | 99.387 | 11121 | 99.392 |
|  | **C - G - T** | 30 | 0.249 | 29 | 0.259 |
|  | **T - G - C** | 13 | 0.108 | 13 | 0.116 |
|  | **T - A - T** | 12 | 0.099 | 11 | 0.098 |
|  | **T - G - G** | 7 | 0.058 | 7 | 0.063 |
|  | **G - G - T** | 7 | 0.058 | 5 | 0.045 |
|  | **T - T - T** | 2 | 0.017 | 2 | 0.018 |
|  | **T - G - A** | 2 | 0.017 | 2 | 0.018 |
|  | **A - G - T** | 1 | 0.008 | -1 | -0.009 |
| P3 | **T - A - A** | 7924 | 98.988 | 7542 | 98.976 |
|  | **T - G - A** | 52 | 0.650 | 49 | 0.643 |
|  | **C - A - A** | 11 | 0.137 | 11 | 0.144 |
|  | **T - A - G** | 4 | 0.050 | 4 | 0.052 |
|  | **T - T - A** | 4 | 0.050 | 4 | 0.052 |
|  | **T - C - A** | 3 | 0.037 | 3 | 0.039 |
|  | **T - A - C** | 2 | 0.025 | 2 | 0.026 |
|  | **T - A - T** | 2 | 0.025 | 2 | 0.026 |
|  | **A - G - A** | 1 | 0.012 | 1 | 0.013 |
|  | **G - A - A** | 1 | 0.012 | 1 | 0.013 |
|  | **T - G - C** | 1 | 0.012 | 1 | 0.013 |
| P5 | **C - A - T** | 9759 | 98.586 | 9177 | 98.635 |
|  | **C - G - T** | 79 | 0.798 | 72 | 0.774 |
|  | **C - A - C** | 33 | 0.333 | 29 | 0.312 |
|  | **C - A - G** | 10 | 0.101 | 9 | 0.097 |
|  | **T - A - T** | 7 | 0.071 | 6 | 0.064 |
|  | **A - A - T** | 3 | 0.030 | 3 | 0.032 |
|  | **C - A - A** | 3 | 0.030 | 3 | 0.032 |
|  | **C - C - T** | 2 | 0.020 | 2 | 0.021 |
|  | **C - T - T** | 1 | 0.010 | 1 | 0.011 |
|  | **G - A - T** | 1 | 0.010 | 1 | 0.011 |
|  | **G - G - T** | 1 | 0.010 | 1 | 0.011 |
| P51 | **C - G - T** | 11628 | 99.614 | 10810 | 99.604 |
|  | **C - A - T** | 16 | 0.137 | 14 | 0.129 |
|  | **C - G - C** | 15 | 0.129 | 15 | 0.138 |
|  | **C - G - G** | 6 | 0.051 | 6 | 0.055 |
|  | **C - G - A** | 3 | 0.026 | 3 | 0.028 |
|  | **T - G - T** | 3 | 0.026 | 3 | 0.028 |
|  | **A - G - T** | 1 | 0.009 | 1 | 0.009 |
|  | **C - C - T** | 1 | 0.009 | 1 | 0.009 |

*Black: vaccine nucleotide, blue: wild-type nucleotide, red: unknown significance

**Supplementary figures**

Supplementary Figure 1:


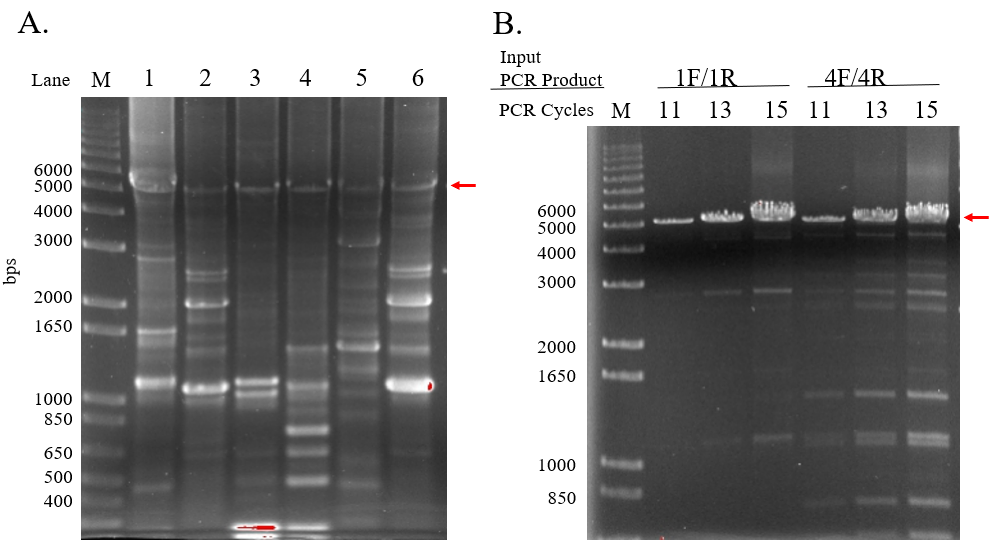


Supplementary Figure 2:


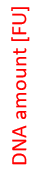

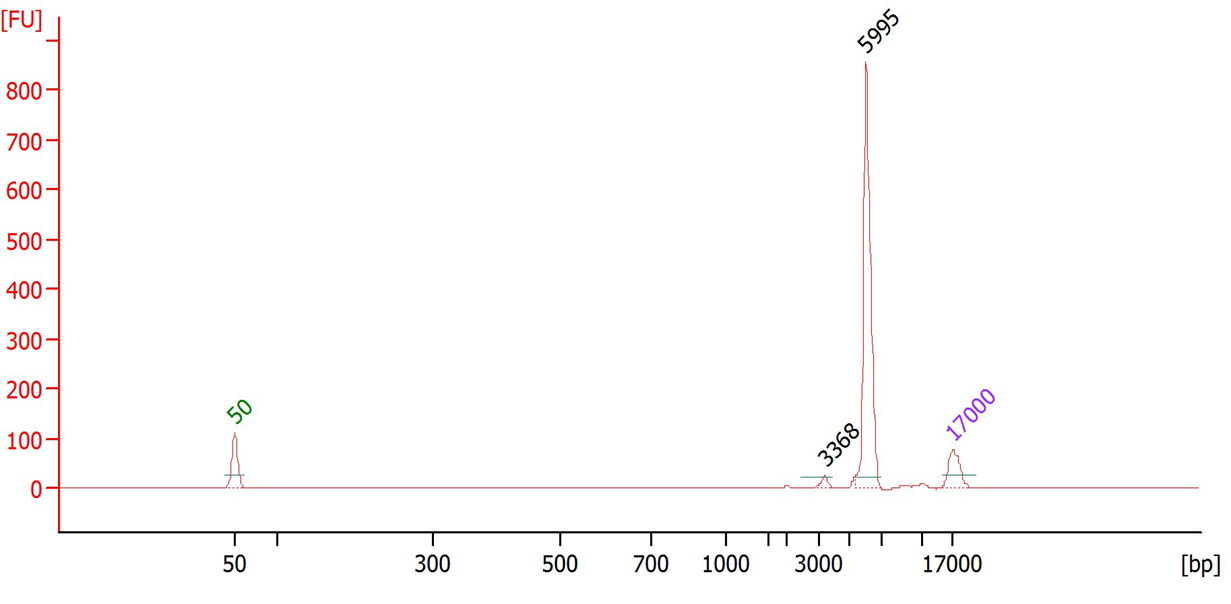

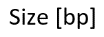


Supplementary Figure 3:


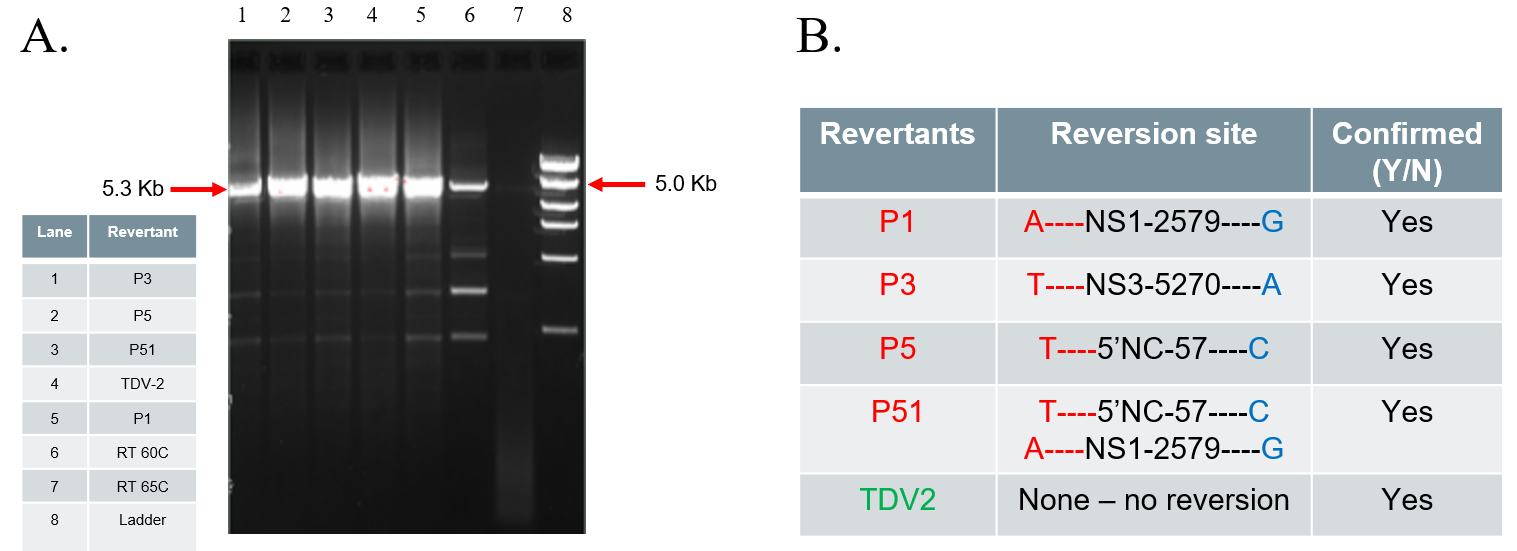


Supplementary Figure 4:


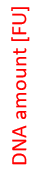

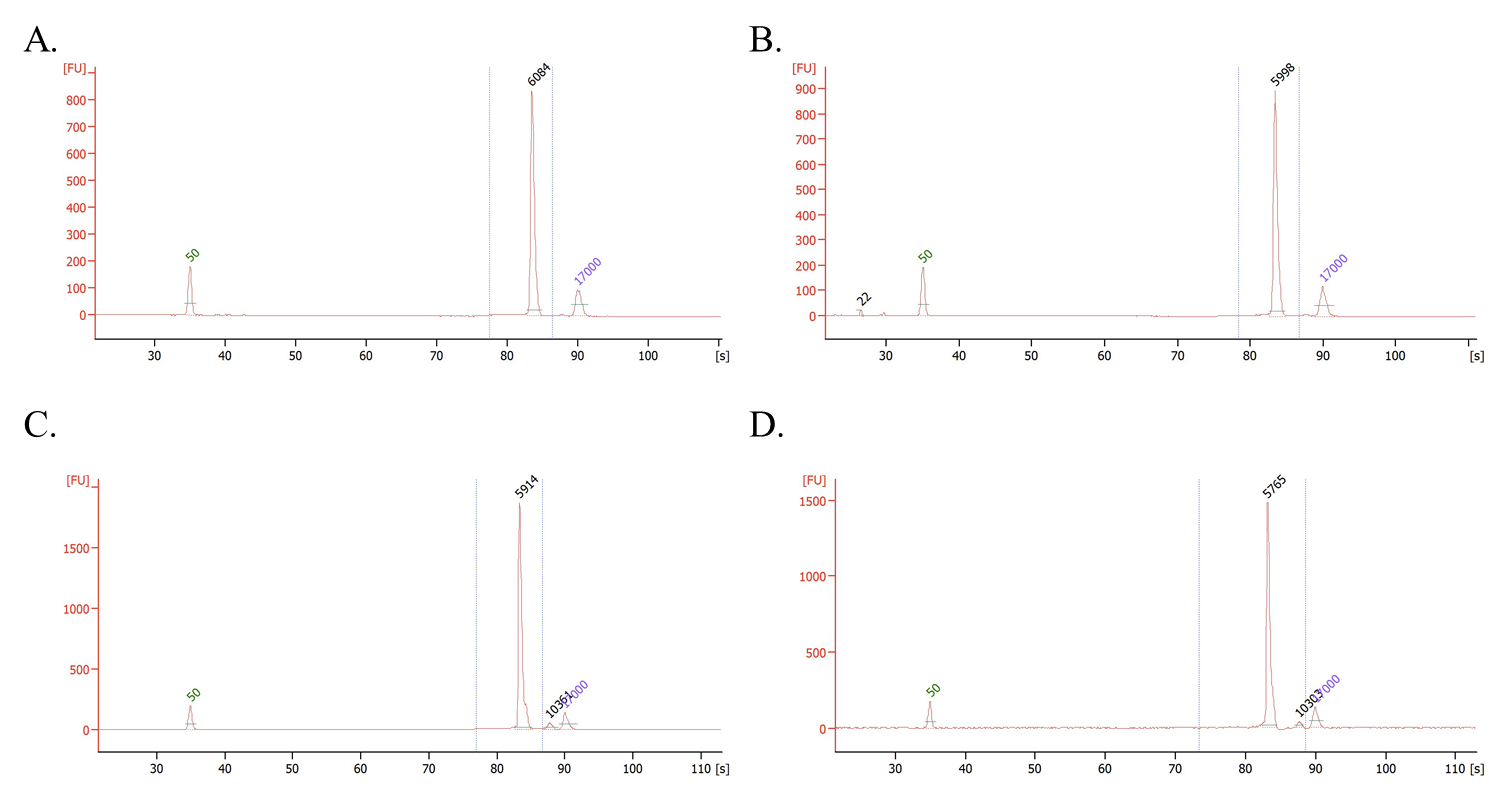

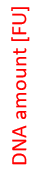

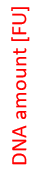

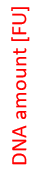

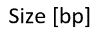

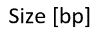

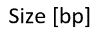

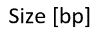


Supplementary Figure 5:


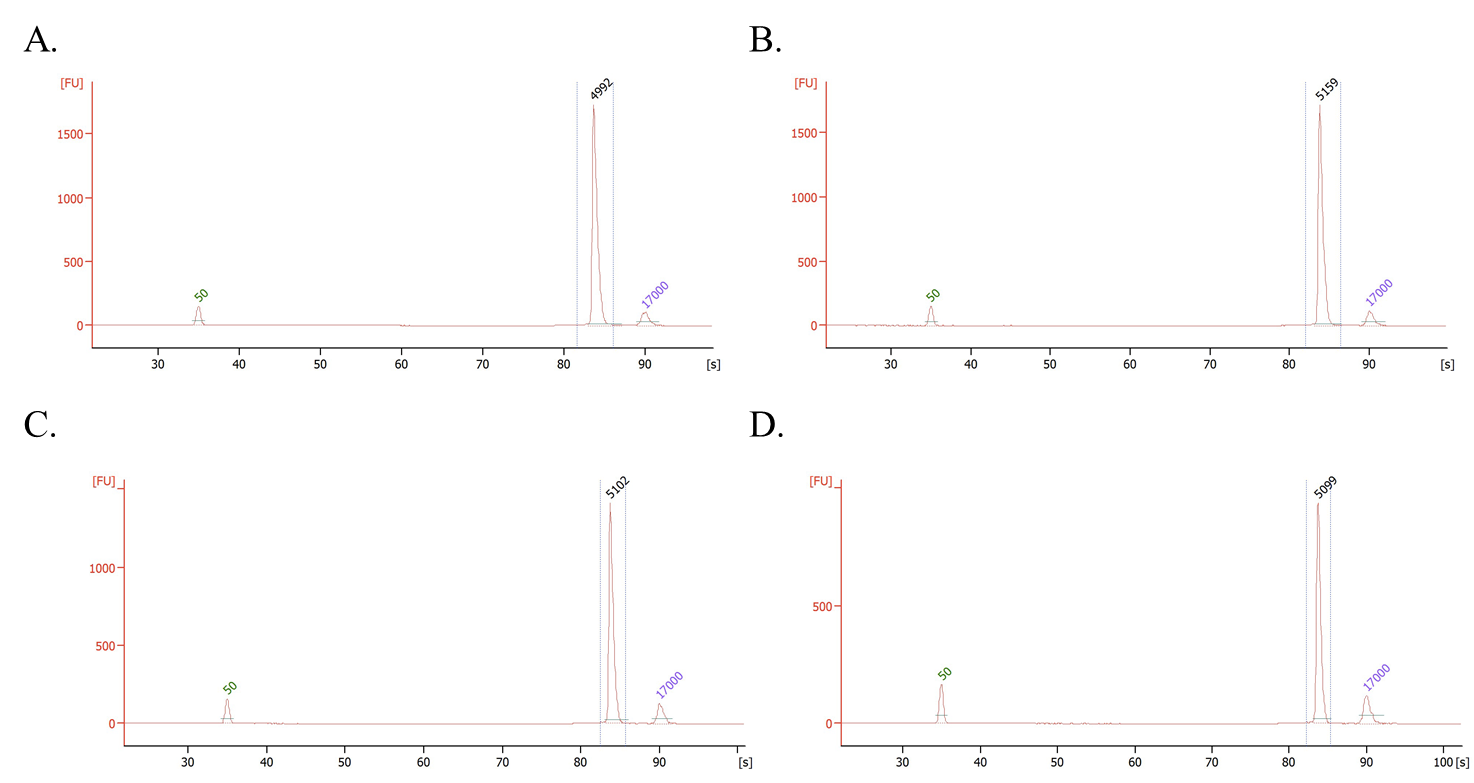

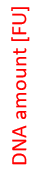

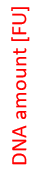

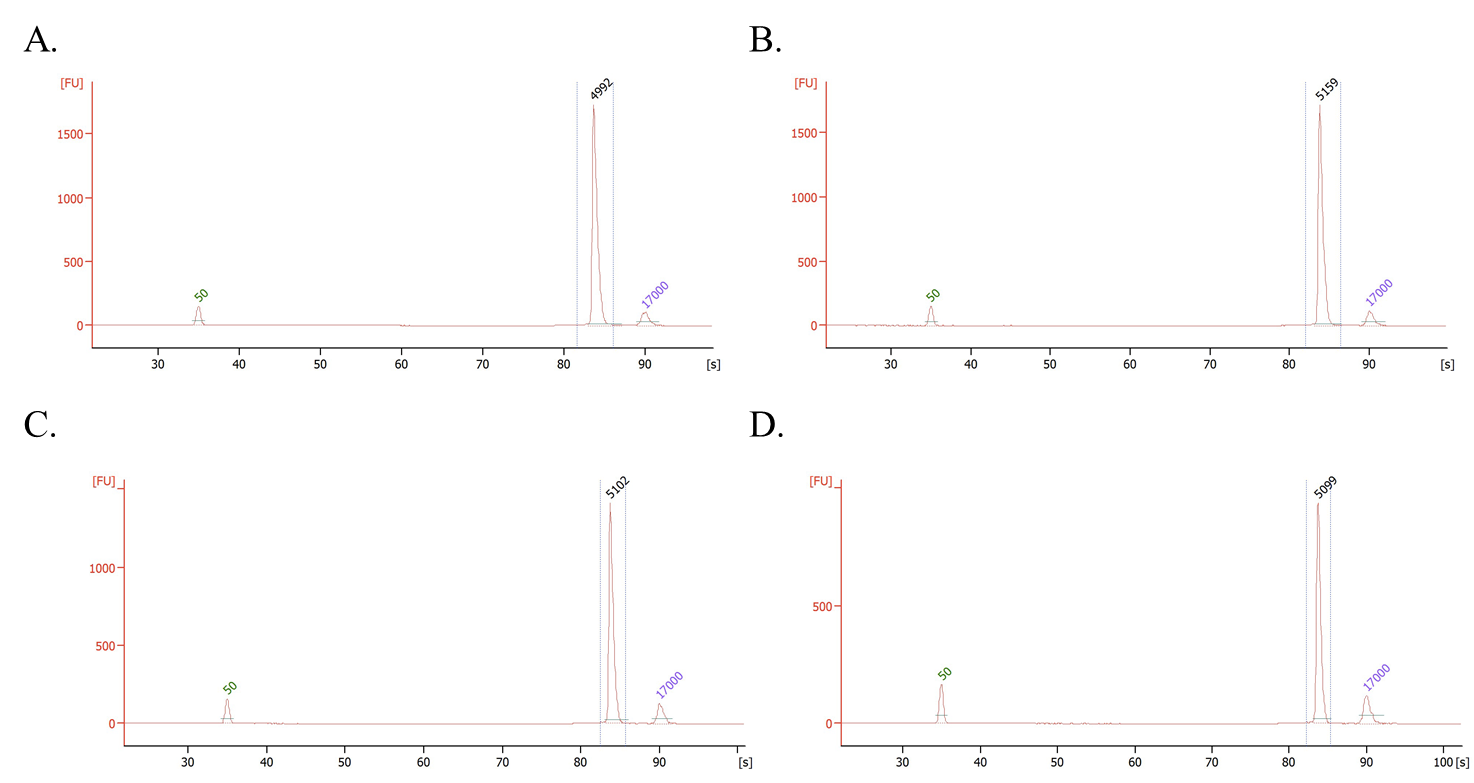

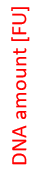

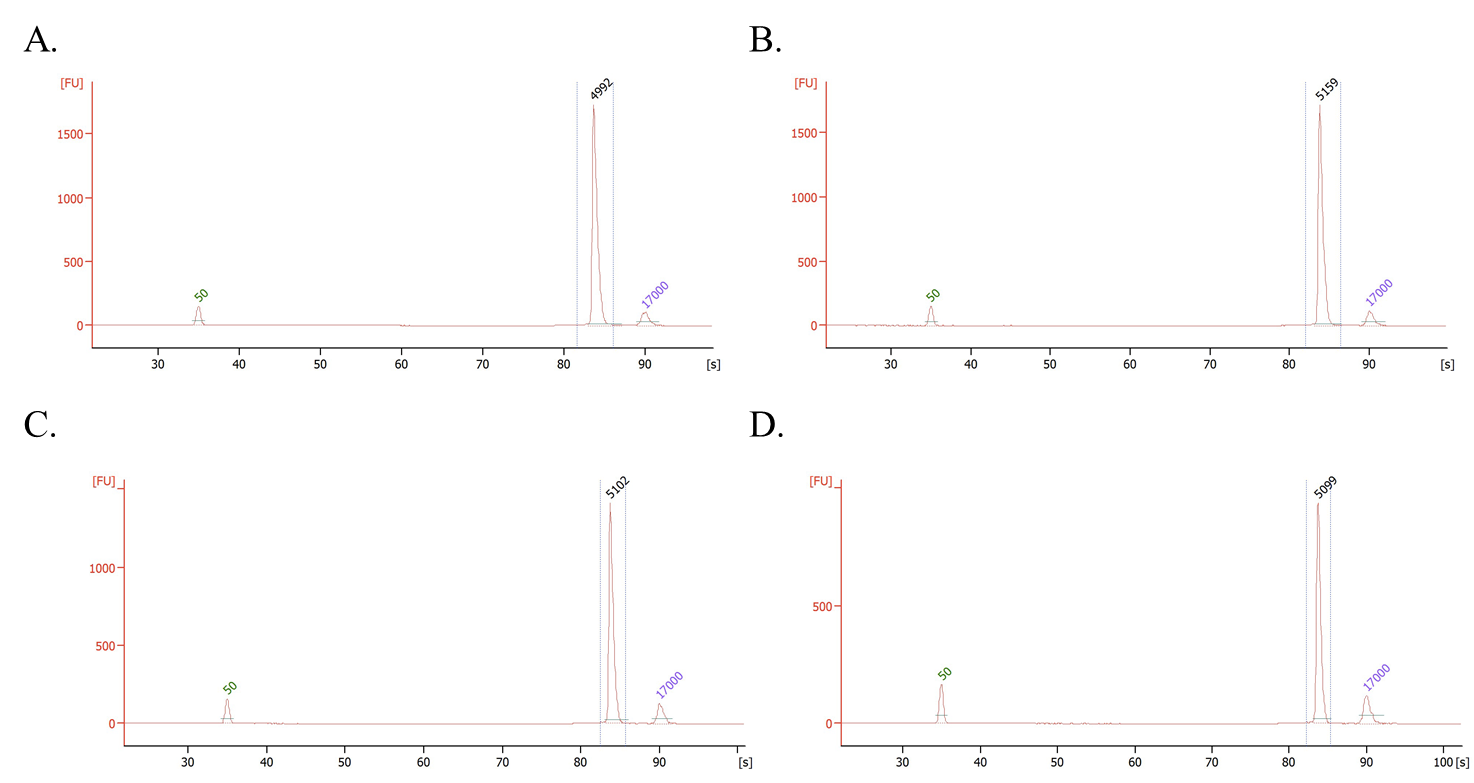

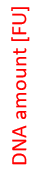

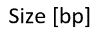

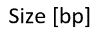

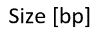

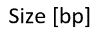

Supplement: bpae004_Supplementary_Data [file bpae004_supplementary_data.zip › Supplementary_figures_and_tables_05_01_2024_submitted.docx]
