# Supplementary material for: Identification and quantitation of multiple variants in RNA virus genomes
Source: Biol Methods Protoc. 2024 Feb 3;9(1):bpae004. doi: 10.1093/biomethods/bpae004 (PMC10898329; doi:10.1093/biomethods/bpae004)
Supplement: bpae004_Supplementary_Data [file bpae004_supplementary_data.zip › SequencingManuscript_Supplementary_Methods_clean_05_01_2024_submitted.docx]

**Supplementary Methods**

**Materials**

- Input RNA. 2.8 x 10^7^ viral RNA copies/μL and a concentration of 20-35 ng/μL
- RT Oligonucleotide (10 μM)
- PCR primers (10 μM)
- SuperscriptIII First-Strand synthesis kit. Thermo Fisher catalog number: 18080051. The kit contains the required reagents RNAseOUT, 10X RT buffer, RNAse H, 25 mM MgCl_2_, and 10 mM dNTPs
- 5 M Betaine stock solution. Affymetrix catalog number: 77507 (or other source)
- Advantage 2 polymerase. Clontech catalog number: 639201 (or other source)
- SMRTbell™ Template Prep Kit. PacBio catalog number: 100-259-100
- DNA/Polymerase Binding Kit. PacBio catalog number: 100-372-700
- MagBead Kit. PacBio catalog number: 100-676-500
- DNA Sequencing Reagent. PacBio catalog number: 100-612-400
- DNA Internal Control Complex. PacBio catalog number: 100-364-600
- SMRT® Cells. PacBio catalog number: 100-171-800
- Pre-washed AMPure XP Beads. Beckman Coulter catalog number: A63881
- Qiagen Buffer (EB). Qiagen catalog number: 19086
- Magnetic rack. ThermoFisher Scientific catalog number: 12321
- DynaMag™-96 Bottom Magnet. ThermoFisher Scientific catalog number: 12332D
- Eppendorf™ DNA LoBind Microcentrifuge Tubes. Eppendorf catalog number: 022431021
- VWR® PCR 8-Well Tube Strips. Catalog number: VWR 20170-004
- Qubit™ dsDNA BR Assay Kit. ThermoFisher Scientific catalog number: Q32850
- Agilent DNA 12000 Kit. Agilent catalog number: 5067-1508
- Hard-Shell PCR Plates 96-well, thin wall. BioRad catalog number: HSP9655

**Quantification of Viral RNAs using RT-qPCR**

The RT-qPCR was performed in a 25 μL volume containing 6.25 μL of 4X TaqMan Fast Virus 1-Step Master Mix (Thermo Fisher Scientific), 0.5 μL of forward primer D2-1929, 20 μM (5’-TCCATGCAAGATCCCTTT TGA-3’), 0.5 μL of reverse primer cD2-2116, 20 μM (5’-TCTTAAACCAGTTGAGCT TCAGTT GT-3’), 0.5 μL of reporter probe VIC2000, 20 μM (5’-/VIC/CAACCCAATTGTGACAGAA/MGBNFQ/-3’), Quencher: NFQ-MBG, 5 μL of viral RNA being tested, and 12.5 μL RNAse-free water. The qRT-PCR was performed using the QuantStudio^TM^ 7 Flex System (Applied Biosciences) with the following cycling conditions: RT at 50 °C for 5 minutes and 95 °C for 20 seconds, followed by 45 cycles of PCR at 95 °C for 3 seconds and 60 °C for 30 seconds.

To quantify the copies of viral genomes, a synthetic standard RNA containing 2.7×10^7^ copies per µL of DENV2 viral RNA was used to establish a standard curve. Standard curves for 10-fold diluted standard RNAs were generated by plotting their cycle threshold numbers (Ct) versus their dilution factors.

**RT-PCR and nested PCR of TDV-2**

RT was carried out on 6.5 μL (139.1 ng) of the TDV-2 viral RNA for each of the reverse primers in Supplementary Table 3 using the Superscript III First-Strand synthesis kit from Thermo Fisher Scientific (catalog number 18080051) that contains RNAse OUT, 10X RT buffer, RNAse H, 25 mM MgCl_2_, and 10 mM dNTPs.

Briefly, 6.5 μL (139.1 ng) of the TDV-2 viral RNA, 1 μL of dNTPs (Final 0.5 mM each) and 1 μL of reverse primer (Final 0.5 μM) was added to 0.2 mL PCR tubes and incubated at 65 °C for 5 minutes in a thermocycler and then placed on ice for 1 minute. Then, 2 μL of 10X RT buffer (Final 1X), 4 μL of 25 mM MgCl_2_ (Final 5 mM), 4 μL of 5 M Betaine (Final 1 M) from Affymetrix (catalog number 77507), 1 μL of RNAseOUT (Final 2 U/μL), and 0.5 μL of Superscript III (Final 5 U/μL) was added to the PCR tubes containing the RNA, dNTPs and primer mixture. The RT mixtures were incubated at 49 °C for 1 hour and then at 85 °C for 5 minutes in a thermocycler. The PCR tubes were placed on ice and then 2 units of RNAse H was added to the RT mixtures and then incubated at 37 °C in a thermocycler. The RT reactions containing first strand cDNA were placed on ice and used for touchdown PCR.

Six primer pairs (Supplementary Table 3 1-6 F/R) were used in touchdown PCR reactions using Advantage 2 polymerase from Clontech (catalog number 639201). For each primer set, 5 μL of cDNA, 5 μL of 10X Advantage 2 PCR buffer (Final 1X), 12 μL of 5 M Betaine (Final 1.2 M), 1 μL of 10 mM dNTPs (Final 200 μM), 1 μL of each primer (Final 200 μM each) from the primer pairs in Supplementary Table 3, 1 μL of 50X Advantage 2 polymerase and 24 μL of PCR grade water were added to new 0.2 mL PCR tubes. The PCR reaction tubes were placed into a thermocycler and touchdown PCR was done as follows: 95 °C for 30 seconds, annealing at 66 °C for 20 seconds with a 1 degree drop each cycle for 10 cycles (i.e., cycle 1: 66 °C, cycle 2: 65 °C, cycle 3: 64 °C...) and extension at 68 °C for 6 minutes. An additional 25 cycles were run at 95 °C for 30 seconds, annealing at 53 °C for 20 seconds and extension at 68 °C for 6 minutes. RT-PCR products were run on 1% agarose gels in 1 X TAE buffer (stained with ethidium bromide) using electrophoresis (Supplementary Figure 1 A).

The PCR products generated using primer pairs 1 and 4 were diluted 10-fold and then used for nested PCR using primers 5F and 3R (Supplementary Table 3) to reduce non-specific amplification. PCR was run as described above except that after the first 10 cycles, the PCR reactions were run for an additional 1, 3, or 5 cycles instead of 25 cycles to determine the number of PCR cycles that showed the lowest amount of smearing, following gel electrophoresis (Supplementary Figure 1 B). The PCR product produced using primer pair 1 (1F/R) showed fewer non-specific bands and was taken forward to annealing temperature optimization to further reduce non-specific amplification. To optimize annealing temperature, nested gradient touchdown PCR was performed as follows: 3.2 μL of the 10-fold diluted PCR product using primer pair 1, 16 μL of 10X Advantage 2 PCR buffer (Final 1X), 38.4 μL of 5 M Betaine (Final 1.2 M), 3.2 μL of 10 mM dNTPs (Final 200 μM), 3.2 μL each of primers 5F and 3R (Final 200 μM each) in Supplementary Table 3, 3.2 μL of 50X Advantage 2 polymerase and 89.6 μL of PCR grade water were added to a 0.5 mL LoBind Eppendorf tube. Then, 10 μL of the PCR reaction mixture was aliquoted to 16 0.2 mL PCR tubes. The PCR reaction tubes were placed into a thermocycler and touchdown PCR was done as follows: 95 °C for 30 seconds, annealing at 66 °C for 20 seconds with a 1 degree drop each cycle for 10 cycles (i.e., cycle 1: 66 °C, cycle 2: 65 °C, cycle 3: 64 °C...) and extension at 68 °C for 6 minutes. An additional 1, 3, or 5 cycles were run at 95 °C for 30 seconds, annealing at 53 °C, 54.2 °C, 56 °C, 58.2 °C, or 60 °C for 20 seconds and extension at 68 °C for 6 minutes; followed by a final extension step at 68 °C for 6 minutes. RT-PCR products were run in 1% agarose gels in 1 X TAE buffer (stained with ethidium bromide) using electrophoresis. Then, the PCR reaction was scaled up as follows: 8 μL of 10-fold diluted PCR (product produced using primer set 1), 40 μL of 10X Advantage 2 PCR buffer (Final 1X), 96 μL of 5 M Betaine (Final 1.2 M), 8 μL of 10 mM dNTPs (Final 200 μM), 8 μL each of primers 5F and 3R (Final 200 nM each) in Supplementary Table 3, 8 μL of 50X Advantage 2 polymerase and 224 μL of PCR grade water were added to a 1.5 mL LoBind Eppendorf tube. Then, 50 μL of the PCR reaction mixture was aliquoted to 8 0.2 mL PCR tubes. The PCR reaction tubes were placed into a thermocycler and touchdown PCR was done as follows: 95 °C for 30 seconds., annealing at 66 °C for 20 seconds with a 1 degree drop each cycle for 10 cycles (i.e., cycle 1: 66 °C, cycle 2: 65 °C, cycle 3: 64 °C...) and extension at 68 °C for 6 minutes. One additional cycle was run at 95 °C for 30 seconds, annealing at 56 °C for 20 seconds and extension at 68 °C for 6 minutes; followed by a final extension step at 68 °C for 6 minutes. The PCR products were pooled into a 1.5 mL LoBind Eppendorf tube and a 0.45X Ampure XP bead purification was used to remove non-specific amplification products less than 3 kilobases Briefly, 0.45 X volumes or 180 μL of Ampure XP beads were added to the pooled PCR product and rotated at room temperature for 20 minutes to bind the DNA to the beads. The supernatant was removed and the DNA bound beads were placed on a magnetic rack and washed twice with 70% ethanol. While still on the magnetic rack, the ethanol was discarded and the beads were dried for 1 minute. The DNA was eluted from the beads by adding 39 μL EB (Qiagen catalog number 19086) and vortexing at 2000 rpm for 2 minutes at room temperature. 37 μL of the eluent was placed into a new 0.2 mL PCR tube for library preparation and another 1 μL was used for fragment analysis using the Bioanalyzer 2100 and the Agilent DNA 12000 Kit (Agilent catalog number 5067-1508) according to the manufacturer’s instructions. The Bioanalyzer electropherogram plot of the amplification product after Ampure bead purification is shown in Supplementary Figure 2.

**PacBio Sequencing and Variant Analysis of Nested PCR**

The material from the optimized RT-PCR reaction (Supplementary Figure 2) was used to construct a PacBio sequencing library according to PacBio’s “Procedure & Checklist - Amplicon Template Preparation and Sequencing” protocol (document number PN 100-801-600-02). The reads were aligned to the DENV reference genome (GenBank U87412.1) using BLASR and variants were called with QUIVER using the PacBio SMRT Analysis Portal, containing components Daemon 2.3.0.139497, SMRTpipe 2.3.0.139497, SMRT Portal 2.3.0.140893, SMRT View 2.3.0.140836. The data are summarized in the Results section (Supplementary Table 4).

**RT-PCR Optimization to Produce a Single TDV-2 PCR Product**

To identify new combinations of PCR primers for optimization, in silico or ePCR with the previously designed primer sets was done, allowing for 30% primer binding site mismatch, with Unipro UGENE software. Based on the ePCR analysis that demonstrated low off target binding and our results with nested PCR, primers 5F and 3R were selected for optimization. Primer sequences are in Supplementary Table 3. Briefly, 6.5 μL (139.1 ng) of the TDV-2 viral RNA, 1 μL of dNTPs (Final 0.5 mM each) and 1 μL of reverse primer 3R (Final 0.5 μM) was added to 0.2 mL PCR tubes and incubated at 65 °C for 5 minutes in a thermocycler and then placed on ice for 1 minute. Then, 2 μL of 10X RT buffer (Final 1X), 4 μL of 25 mM MgCl_2_ (Final 5 mM), 4 μL of 5M Betaine (Final 1 M) from Affymetrix (catalog number 77507), 1 μL of RNAseOUT (Final 2 U/μL), and 0.5 μL of Superscript III (Final 5 U/μL) was added to the PCR tubes containing the RNA, dNTPs and primer mixture. The RT mixtures were incubated at 50 or 55 °C for 1 hour and then at 85 °C for five minutes in a thermocycler. The PCR tubes were placed on ice and then 2 units of RNAse H was added to the RT mixtures and then incubated at 37 °C in a thermocycler. The RT reactions containing first strand cDNA were placed on ice and used for touchdown PCR.

Primers 5F and 3R (Supplementary Table 3) were used in touchdown PCR reactions using Advantage 2 polymerase from Clontech (catalog number 639201). 5 μL of cDNA, 5 μL of 10X Advantage 2 PCR buffer (Final 1X), 12 μL of 5M Betaine (Final 1.2M), 1 μL of 10 mM dNTPs (Final 200 μM), 1 μL of each primer (Final 200 μM each) 5F and 3R, 1 μL of 50X Advantage 2 polymerase and 24 μL of PCR grade water were added to a new 0.2 mL PCR tube. 5 μL of PCR reaction was aliquoted to seven 200 μL PCR tubes, one for each annealing temperature described below. The PCR reaction tubes were placed into a thermocycler and touchdown PCR was done as follows: one cycle: 95 °C for 1 minute; 10 cycles: 95 °C for 30 seconds, 68-58 °C for 20 seconds (1 °C decrease every cycle for 10 cycles), 68 °C for 6 minutes; 5 cycles: 95 °C for 30 seconds, 53, 55.4, 57.2, 59.4, 61.9, 65.7, or 68 °C for 20 seconds and 68 °C for 6 minutes; one cycle: 68 °C for 6 minutes.

RT-PCR was performed by mixing 6.5 μl of TDV2 viral RNA (~10^8^ viral copies/μL) as described above. Following RT-PCR, 5 μL of product was run in a 1% TAE agarose gel using electrophoresis (Figure 1).

**Introduction of Unique Molecular Identifiers**

A fully random 16 nucleotide UMI and a 5’ PCR Primer IIA binding site were added to the 5’ end of the 3R primer. Primer sequences and characteristics are shown in Supplementary Table 3. RT-PCR was performed with the optimized conditions, using 6.5 μL of TDV2 viral RNA (~10^8^ viral copies/μL) as described above. Specifically, RT was performed at 55 °C for one hour instead of 50 °C. A touchdown PCR reaction was set up as described above and was run with the following cycling parameters: One cycle: 95 °C for 1 minute; 10 cycles: 95 °C for 30 seconds, 68-58 °C for 20 seconds (1 °C decrease every cycle for 10 cycles), 68 °C for 6 minutes; 5 cycles: 95 °C for 30 seconds, 57 °C for 20 seconds, 68 °C for 6 minutes; one cycle: 68 °C for 6 minutes. 5 μL of the PCR product was run in a 1% TAE agarose gel stained with ethidium bromide (Figure 2 A). Subsequently, large-scale PCR, in which the reaction volume was scaled up 3X, was performed. The initial PCR product (45 μL) was combined with the large-scale PCR reaction and a 0.45X (87.75 μL) AMPure XP bead purification was used to concentrate the PCR product and remove excess primers. A 1 μL aliquot of the product was used from fragment analysis using the Bioanalyzer 2100 and the Agilent DNA 12000 kit (Figure 2 B). The remaining PCR product was used to synthesize Pacbio sequencing libraries as described below.

**PacBio Amplicon Template Preparation and Sequencing Protocol**

AMPure XP bead washes are necessary before using AMPure XP beads. The polymer coating on the beads can leak into the storage solution and interfere with PacBio polymerase binding. Agencourt AMPure XP beads were thoroughly resuspended and 500 μL was pipetted into 1.5 mL LoBind Eppendorf tubes. The beads were centrifuged for 1 minute at max speed in a benchtop microcentrifuge and placed on a magnetic rack until the supernatant cleared. The supernatant was transferred to another 1.5 mL LoBind Eppendorf tube. 1 mL of molecular biology grade water was added to the beads and vortexed to re-suspend the beads completely. The beads were centrifuged for 1 minute at maximum speed in a benchtop microcentrifuge and bead containing tubes were placed on a magnetic rack until the supernatant cleared. The water was discarded and the beads were washed four more times with water. After the fourth wash 1 mL of Qiagen EB was added to the bead pellet and vortexed to completely re-suspend the beads. The bead mixture was centrifuged for 1 minute at max speed in a benchtop microcentrifuge and then placed on a magnetic rack until the supernatant cleared. The EB was discarded and the beads were completely re-suspended in the original AMPure XP supernatant. The cleaned beads were stored at 4 °C for up to 3 months or until used.

The large-scale PCR reaction was pooled into a 1.5 mL LoBind Eppendorf tube and 0.45 X volumes of cleaned AMPure XP beads were added to the PCR reaction and mixed thoroughly. The mixture was spun down to collect the beads and then rotated for 10 minutes at room temperature to allow the DNA to bind to the beads. After rotating, the bead/DNA mixture was centrifuged for 1-5 seconds and placed on a magnetic bead rack until the beads collected to the side of the tubes and the solution appeared clear. The actual time required to collect the beads to the side depends on the volume of beads added. With the tubes still on the magnetic bead rack, the supernatant was slowly pipetted off without disturbing the bead pellet. The beads were washed by slowly dispensing 1.5 mL of freshly prepared 70% ethanol against the side of the tube opposite the beads. The beads were incubated in ethanol for 30 seconds without disturbing the bead pellet and then the ethanol was discarded. The ethanol wash was repeated. The bead tubes were centrifuged for 5 seconds and placed back on a magnetic bead rack. The residual ethanol was removed by pipetting. The tubes were removed from the magnetic bead rack and allowed to air-dry with the tube caps open for 30 to 60 seconds. 39 μL of Qiagen EB was added to the beads and mixed by vortexing for 1 to 2 minutes at 2000 rpm until homogeneous. The bead mixture was spun briefly to pellet the beads and then placed on a magnetic rack until the supernatant cleared. 37 μL of the eluate was collected into a new 200 μL PCR tube and placed on ice.

The eluted DNA was used for the DNA damage repair step of the “Procedure & Checklist - Amplicon Template Preparation and Sequencing” protocol (document number PN 100-801-600-02). 5 μL of DNA Damage Repair Buffer, 0.5 μL NAD+, 5 μL ATP high, 0.5 μL dNTP, and 2 μL of DNA Damage Repair Mix from the SMRTbell™ Template Prep Kit was added to the DNA and mixed by flicking the tube. The mixture was centrifuged quickly to collect the reaction in the bottom of the tube. The sample tube was placed in a thermocycler and incubated at 37 °C for 1 hour and then at 4 °C for 1 minute and then put on ice.

DNA end repair was performed by adding 2.5 μL End Repair Mix and the sample was mixed by flicking the tube. The sample was centrifuged briefly, placed in a thermocycler and incubated at 25 °C for 5 minutes and then placed back on ice. The end-repaired reaction was cleaned up using 0.45 X volumes of AmpureXP beads as described above and eluted in 33 μL of Qiagen EB. 31 μL of eluate was collected into a new 200 μL PCR tube and placed on ice.

SMRTbell adapters were ligated to the end-repaired DNA by adding 2 μL of Blunt Adapter, 4 μL of Template Prep Buffer, 2 μL of ATP Lo, and 1 μL of Ligase. The sample was mixed by flicking the reaction tube and centrifuged briefly. The sample was placed in a thermocycler, incubated at 25 °C for 1 hour and then at 65 °C for 10 minutes to inactivate the ligase and placed on ice. 0.5 μL of Exo III and 0.5 μL ExoVII was added to the sample and incubated at 37°C for 1 hour in a thermocycler.

The SMRTBell DNA library was cleaned up using 0.45 X volumes of AMPure XP beads, as described above and eluted in 52 μL of Qiagen EB. 50 μL of eluate was collected into a new 200 μL PCR tube and a second round of clean up was performed using 0.45 X volumes of AMPure XP beads. The library was eluted in 12 μL of EB. 10 μL of eluate was collected and stored at -20 °C until used for sequencing. RT-PCR and library preparation was performed as described above for subsequent TDV-2 revertant samples and complex TDV-2 revertant mixtures.

**Anneal, Bind and Sequence SMRTbell Libraries**

The PacBio P6C4 Binding Calculator, version 2.3.1.1, was used to determine the conditions for annealing the sequencing primer and binding polymerase to SMRTbell libraries. Please see the Binding Calculator parameters below:

| **Sample Name** | **TDV-2** | **P1** | **P3** | **P5** | **P51** | **Complex Mix 1** | **Complex Mix 2** | **Complex Mix 3** | **Complex Mix 4** |
| --- | --- | --- | --- | --- | --- | --- | --- | --- | --- |
| Sample Volume to Use | 3 μL | 3 μL | 3 μL | 3 μL | 3 μL | 5 μL | 3 μL | 3 μL | 3 μL |
| Concentration | 10.4 ng/μL | 20.2 ng/μL | 50.8 ng/μL | 63.8 ng/μL | 40.6 ng/μL | 12.8 ng/μL | 33.4 ng/μL | 21.2 ng/μL | 24 ng/μL |
| Insert size | 10000 bp | 10000 bp | 10000 bp | 10000 bp | 10000 bp | 10000 bp | 10000 bp | 10000 bp | 10000 bp |
| Size Selection | No | No | No | No | No | No | No | No | No |
| Protocol | MagBead OCPW | MagBead OCPW | MagBead OCPW | MagBead OCPW | MagBead OCPW | MagBead OCPW | MagBead OCPW | MagBead OCPW | MagBead OCPW |
| Binding Kit | P6 | P6 | P6 | P6 | P6 | P6 | P6 | P6 | P6 |
| Preparation | Small | Small | Small | Small | Small | Small | Small | Small | Small |
| Long-Term Storage | No | No | No | No | No | No | No | No | No |
| DNA Control | No | No | No | No | No | No | No | No | No |
| Complex Reuse | No | No | No | No | No | No | No | No | No |
| Standard Concentration | Yes | Yes | Yes | Yes | Yes | Yes | Yes | Yes | Yes |
| Concentration On Plate | Custom (0.015 nM) | Custom (0.0125 nM) | Custom (0.0125 nM) | Custom (0.0125 nM) | Custom (0.0125 nM) | Custom (0.015 nM) | Custom (0.015 nM) | Custom (0.015 nM) | Custom (0.015 nM) |
| Control To Template | Default (1.2%) | Default (1.2%) | Default (1.2%) | Default (1.2%) | Default (1.2%) | Default (1.2%) | Default (1.2%) | Default (1.2%) | Default (1.2%) |
| Polymerase:Template Ratio | Default (10) | Default (10) | Default (10) | Default (10) | Default (10) | Default (10) | Default (10) | Default (10) | Default (10) |
| Primer:Template Ratio | Default (20) | Default (20) | Default (20) | Default (20) | Default (20) | Default (20) | Default (20) | Default (20) | Default (20) |

The 1 μL of sequencing primer and 32.3 μL of elution buffer was added to a new 200 μL PCR tube and incubated at 80 °C for 2 minutes and then placed on ice. The diluted sequencing primer was annealed to the template by adding the appropriate amount of reagents in the order listed below to a new 200 μL PCR tube.

| **Sample Name** | **TDV-2** | **P1** | **P3** | **P5** | **P51** | **Complex Mix 1** | **Complex Mix 2** | **Complex Mix 3** | **Complex Mix 4** |
| --- | --- | --- | --- | --- | --- | --- | --- | --- | --- |
| Volume H_2_0 | 2.6 μL | 5.8 μL | 19.2 μL | 24.9 μL | 14.7 μL | 4.3 μL | 11.6 μL | 6.3 μL | 7.5 μL |
| 10x Primer Buffer | 0.96 μL | 1.1 μL | 2.8 μL | 3.5 μL | 2.2 μL | 1.2 μL | 1.8 μL | 1.2 μL | 1.3 μL |
| Library Sample Volume | 5 μL | 3 μL | 3 μL | 3 μL | 3 μL | 5 μL | 3 μL | 3 μL | 3 μL |
| Diluted Sequencing Primer | 1.1 μL | 1.2 μL | 3.1 μL | 3.9 μL | 2.5 μL | 1.3 μL | 2.1 μL | 1.3 μL | 1.5 μL |
| Total Volume | 9.6 μL (at 0.8333 nM) | 11.2 μL (at 0.8333 nM) | 28.1 μL (at 0.8333 nM) | 35.3 μL (at 0.8333 nM) | 22.5 μL (at 0.8333 nM) | 11.8 μL (at 0.8333 nM) | 18.5 μL (at 0.8333 nM) | 11.7 μL (at 0.8333 nM) | 13.3 μL (at 0.8333 nM) |

The mixtures were incubated at 20 °C for 30 minutes and placed on ice. The SA-DNA polymerase was diluted 1:10 in Binding Buffer v2 and placed on ice. dNTPs, DTT, Binding Buffer v2 and diluted SA-DNA polymerase were added to the primer annealed libraries as shown in the table below. The libraries were mixed by flicking the tubes, centrifuged briefly and incubated at 30 °C for 30 minutes and then placed back on ice.

| **Sample Name** | **TDV-2** | **P1** | **P3** | **P5** | **P51** | **Complex Mix 1** | **Complex Mix 2** | **Complex Mix 3** | **Complex Mix 4** |
| --- | --- | --- | --- | --- | --- | --- | --- | --- | --- |
| dNTP | 1.6 | 1.9 μL | 4.7 μL | 5.9 μL | 3.7 μL | 2 μL | 3.1 μL | 2 μL | 2.2 μL |
| DTT | 1.6 | 1.9 μL | 4.7 μL | 5.9 μL | 3.7 μL | 2 μL | 3.1 μL | 2 μL | 2.2 μL |
| Binding Buffer v2 | 1.6 | 1.9 μL | 4.7 μL | 5.9 μL | 3.7 μL | 2 μL | 3.1 μL | 2 μL | 2.2 μL |
| Polymerase Dilution | 1.6 | 1.9 μL | 4.7 μL | 5.9 μL | 3.7 μL | 2 μL | 3.1 μL | 2 μL | 2.2 μL |
| Total Volume | 16 μL (at 0.5 nM) | 18.6 μL (at 0.5 nM) | 46.9 μL (at 0.5 nM) | 58.9 μL (at 0.5 nM) | 37.5 μL (at 0.5 nM) | 19.7 μL (at 0.5 nM) | 30.8 μL (at 0.5 nM) | 19.6 μL (at 0.5 nM) | 22.2 μL (at 0.5 nM) |

The SMRTBell libraries were prepared for sequencing by mixing MagBead Binding Buffer and polymerase bound library in a new 200 μL PCR tube as shown in the table below, the libraries were then placed back on ice.

| **Sample Name** | **TDV-2** | **P1** | **P3** | **P5** | **P51** | **Complex**  **Mix 1** | **Complex**  **Mix 2** | **Complex**  **Mix 3** | **Complex**  **Mix 4** |
| --- | --- | --- | --- | --- | --- | --- | --- | --- | --- |
| MagBead Binding Buffer | 8.7 μL | 8.8 μL | 8.8 μL | 8.8 μL | 8.8 μL | 8.7 μL | 8.7 μL | 8.7 μL | 8.7 μL |
| Polymerase  Bound Library | 0.3 μL | 0.22 μL | 0.22 μL | 0.22 μL | 0.22 μL | 0.3 μL | 0.3 μL | 0.3 μL | 0.3 μL |

MagBeads were mixed until homogenous. For each sample, 35 μL of MagBeads were added to empty 1.5 mL LoBind tubes. The beads were centrifuged for a couple of seconds and placed on a magnetic bead rack until the supernatant cleared. The supernatant was discarded and 35 μL of MagBead Binding Buffer was added to the beads and mixed by pipetting 10 times. The beads were centrifuged for a couple of seconds to collect the beads and placed on a magnetic bead rack until the supernatant cleared and then the supernatant was discarded. 9 μL of diluted library was added to the corresponding bead tube. The bead tube containing the library was mixed until homogenous by flicking the tube and then centrifuged for a couple of seconds to collect the beads and then placed back on ice. The bead library mixture was incubated at 4 °C on a rotator for 30 minutes and then placed back on ice. While the library bead mixture was incubating, the DNA Internal Control Complex was diluted 1:5000 with MagBead Binding Buffer in a new 200 μL PCR tube. After incubation the library bead mixture was centrifuged for a couple of seconds and then placed on a magnetic bead rack until the supernatant cleared. The supernatant was then discarded and the beads placed back on ice. 18 μL of MagBead Binding buffer was added to the beads and mixed by flicking the tubes and placed on a magnetic bead rack until the supernatant cleared. The supernatant was discarded and the beads were washed a second time with MagBead Binding buffer. 3 μL of the diluted Control Mix and 42 μL of MagBead Binding buffer were added to the beads and mixed by flicking the tubes, centrifuged for a couple of seconds to collect the bead mixture and placed on ice until use. A new 96-well PCR plate was placed on ice and the beads from the previous step were mixed by pipetting and 45 μL of the bead mixture was added to an empty well of the 96-well PCR plate. The PCR Plate containing the sequencing libraries, DNA Sequencing Reagents and SMRTCells were loaded into the PacBio RS II. The libraries were sequenced for 360 minutes. The PacBio run was configured using PacBio RS Remote software, version 2.3. The following settings were used: Collection Protocol = MagBead OneCellPerWell v1, AcquisitionTime (Movie Time)=360, InsertSize=10000, StageHS=True, SizeSelectionEnabled=False, DNAControlComplex=2kb_control, Use2ndLook=False, NumberOfCollections=1

**Quantify TDV-2 Variants Using Read Based and UMI Based Methods**

Briefly, the raw sequencing data and DENV reference genome (GenBank accession U87412.1; DENV type 2, Strain 16681) were imported into Smrtanalysis software version 2.3.0.140936.p5.167094. The TDV2, P1, P3, P5, P51, and Complex Mix 1-4 reads were aligned to the DENV reference genome using the RS_ReadsOfInsert_Mapping.1 protocol of the Smrtanalysis software with the default mapping parameters and the following filtering parameters, Minimum Full Passes = 3, Minimum Predicted Accuracy = 90, Minimum Read Length of Insert = 5000, Maximum Read Length of Insert = 7000. The resulting reads of insert fastq file was used in this workflow and consisted of trimming off the UMI 3R adapter sequence, extracting the UMI sequence and appending it to read IDs of the TDV2 read of insert fastq file, aligning the trimmed fastq file to the DENV reference genome using BWA-mem and counting the revertants at each attenuation site using the Sam2Tsv (<http://lindenb.github.io/jvarkit/Sam2Tsv.html> and custom bash and perl scripts. The variant counts using reads and UMIs are shown in Table 1.

**Large-scale PCR to Incorporate UMIs into Complex Revertant Mixtures**

Four complex revertant mixtures (Complex Mix 1- 4) were pooled based on genome copy as determined by qRT-PCR and shipped to NCGR without disclosing any information on the composition. Details of the pools are described in Supplementary Table 2. RT was performed on four complex revertant mixtures, Complex Mix 1-4, as described in the above using 6.5 μL of viral RNA (~ 2.8 x 10^7^ viral copies/μL) and the UMI 3R primer. Next, large-scale touchdown PCR was performed as described above. 5 μL aliquots of each Complex mixture, 1-4, were run in a 1 % TAE agarose gel using electrophoresis (Figure 3 B). Detailed descriptions of PacBio library preparation protocols are described above.

**RT-PCR Optimization and Sanger sequencing to confirm the identity of TDV-2 Revertants**

RT-PCR was performed on TDV-2, and TDV-2 revertants (P1, P3, P5, P51) using Titan One tube RT-PCR kit (Roche) along with Primers D2-1 (Forward): AGTTGTTAGTCTACGTGGACCGAC and cD2-5358 (Reverse): GAAATGGGCTTCGTCCATGATAATCAGG. Optimized RT-PCR conditions were applied to four TDV-2 revertants to amplify a 5.3 kb product using 100 ng viral RNA. The RT-PCR was done as follows: 50 °C for 30 seconds, 94 °C for 2 minutes (1 cycle), denaturation at 94 °C for 15 seconds, annealing at 60 °C for 30 seconds and extension at 68 °C for 5 minutes (30 cycles) and final extension at 68 °C for 5 minutes.

RT-PCR reactions were run in 0.8 % agarose TAE gels (stained with ethidium bromide) using electrophoresis and bands of ~ 5.3 kb were excised from the gel and purified using Qiagen’s Gel extraction kit. 2 µl of 10 X diluted gel extracted PCR product was loaded per lane on a 0.8% agarose gel run in 1 X TAE buffer using electrophoresis (Supplementary Figure 3 A).

Purified PCR products were sent to Wyzer Biosciences LLC for sanger sequencing and sequencing data files were edited and aligned to TDV-2 reference sequencing using DNASTAR Navigator 14 software. The primers used in sanger sequencing reactions and the positions of these primers on TDV-2 genome covering three attenuation loci of interest are in Supplementary Table 5 and the results are summarized in Supplementary Figure 3 B.
